# Synthesis of Functionalized Thiophene Based Pyrazole Amides via Various Catalytic Approaches: Structural Features through Computational Applications and Nonlinear Optical Properties

**DOI:** 10.3390/molecules27020360

**Published:** 2022-01-07

**Authors:** Iram Kanwal, Nasir Rasool, Syeda Huda Mehdi Zaidi, Zainul Amiruddin Zakaria, Muhammad Bilal, Muhammad Ali Hashmi, Adeel Mubarik, Gulraiz Ahmad, Syed Adnan Ali Shah

**Affiliations:** 1 Department of Chemistry, Government College, University Faisalabad, Faisalabad 38000, Pakistan; iramchemist117@gmail.com (I.K.); muhammadbilalgcuf@gmail.com (M.B.); adeelmubarik2@gmail.com (A.M.); gulchemist35@gmail.com (G.A.); 2 Department of Chemistry, Division of Science & Technology, University of Education, Lahore 54770, Pakistan; hudazaidi36@gmail.com (S.H.M.Z.); muhammad.hashmi@ue.edu.pk (M.A.H.); 3 Department of Biomedical Sciences, Faculty of Medicine and Health Sciences, Universiti Malaysia Sabah, Jalan UMS, Kota Kinabalu 88400, Malaysia; 4 Faculty of Pharmacy, Universiti Teknologi MARA Cawangan Selangor Kampus Puncak Alam, Bandar Puncak Alam, Selangor D.E., Puncak Alam 42300, Malaysia; syedadnan@uitm.edu.my; 5 Atta-ur-Rahman Institute for Natural Products Discovery (AuRIns), Universiti Teknologi MARA Cawangan Selangor Kampus Puncak Alam, Bandar Puncak Alam, Selangor D.E., Puncak Alam 42300, Malaysia

**Keywords:** pyrazole, amides, arylation, palladium, cross-coupling, computational

## Abstract

In the present study, pyrazole-thiophene-based amide derivatives were synthesized by different methodologies. Here, 5-Bromothiophene carboxylic acid (**2**) was reacted with substituted, unsubstituted, and protected pyrazole to synthesize the amide. It was observed that unsubstituted amide (5-bromo-*N*-(5-methyl-1*H*-pyrazol-3-yl)thiophene-2-carboxamide (**7**) was obtained at a good yield of about 68 percent. The unsubstituted amide (**7)** was arylated through Pd (0)-catalyzed Suzuki–Miyaura cross-coupling, in the presence of tripotassium phosphate (K_3_PO_4_) as a base, and with 1,4-dioxane as a solvent. Moderate to good yields (66–81%) of newly synthesized derivatives were obtained. The geometry of the synthesized compounds (**9a**–**9h**) and other physical properties, like non-linear optical (NLO) properties, nuclear magnetic resonance (NMR), and other chemical reactivity descriptors, including the chemical hardness, electronic chemical potential, ionization potential, electron affinity, and electrophilicity index have also been calculated for the synthesized compounds. In this study, DFT calculations have been used to investigate the electronic structure of the synthesized compounds and to compute their NMR data. It was also observed that the computed NMR data manifested significant agreement with the experimental NMR results. Furthermore, compound (**9f**) exhibits a better non-linear optical response compared to all other compounds in the series. Based on frontier molecular orbital (FMO) analysis and the reactivity descriptors, compounds (**9c**) and (**9h**) were predicted to be the most chemically reactive, while (**9d**) was estimated to be the most stable among the examined series of compounds.

## 1. Introduction

**Figure 1:**
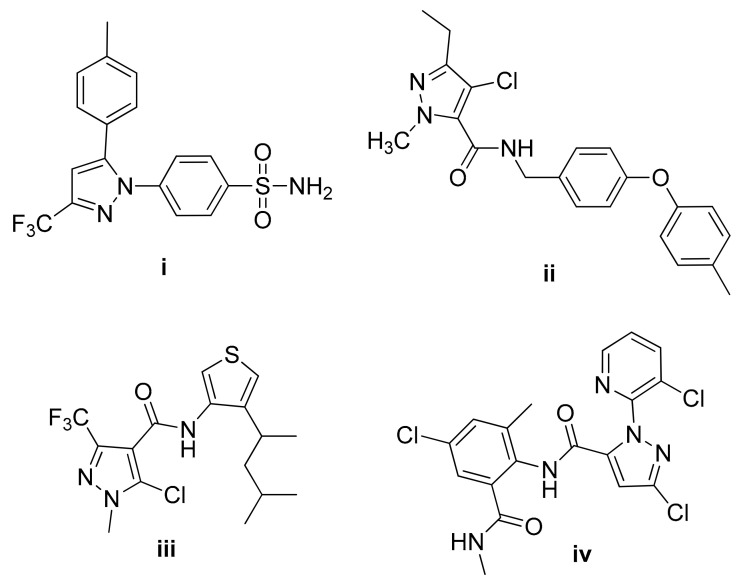
Pyrazole based commercially available drugs (rimonabant (**i**), tolfenpyrad (**ii**), penthiopyrad (**iii**), and chlorantraniliprole (**iv**).

Pyrazole and its derivatives have attracted enormous attention from chemists, as well as from biologists. Compounds having pyrazole and pyrazole amide moieties are well established in the literature as important, biologically active, heterocyclic compounds [1]. The naturally occurring drugs containing pyrazole and pyrazole amide moieties manifest anti-diabetic, anti-tumor, and anti-viral properties [2]. These derivatives are the subject of many research studies, due to their vast biological potential and commercial availability as anti-inflammatory drugs, COX-2 inhibitors, alcohol dehydrogenase inhibitors, and obesity treatments (e.g., rimonabant (**i**)) [3]. Recently, several pyrazole amide derivatives have been developed and commercialized as pesticides, such as tolfenpyrad (**ii**), penthiopyrad (**iii**), and chlorantraniliprole (**iv**) [4] (Figure 1).

Furthermore, Peter Langer and his co-workers reported the direct arylation of pyrazole via Suzuki–Miyaura cross-coupling [5]. Moreover, our research group reported the direct arylation of thiophene and discussed the antibacterial and anti-urease activity of 2-aryl-3-methyl-5-arylthiophenes [6,7]. Previously, the arylation and biological activities of thiophene amide derivatives were also reported by our research group [8]. To the best of our knowledge, the synthesis and arylation of pyrazole-thiophene-based amides have not been reported so far. Therefore, keeping in mind the importance of pyrazole and thiophene moieties, we preliminary reported the results related to the synthesis of pyrazole-thiophene amide and their arylation. The pyrazole-thiophene amide was synthesized via different methodologies and arylated via Suzuki–Miyaura cross-coupling. Furthermore, computational investigation of the newly synthesized compounds was also performed to gain insight into the structural and spectroscopic features of the compounds. DFT calculations on the synthesized compounds have led to a deeper understanding of their geometry and other physical properties, like NLO properties, NMR, and other chemical reactivity descriptors, including the electronic chemical potential, chemical hardness, electron affinity, ionization potential, and electrophilicity index. We have also predicted the NMR spectra of the compounds that could not be isolated.

## 2. Results and Discussion

### 2.1. Chemistry

**Scheme 1:**
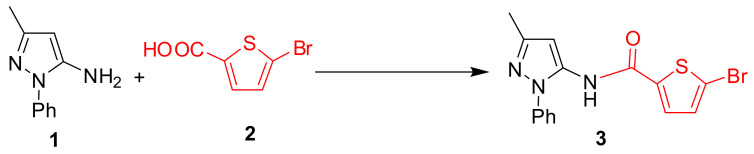
Synthesis of 5-bromo-*N*-(3-methyl-1-phenylpyrazol-5-yl)thiophene-2-carboxamide.

In the present study, the pyrazole amide derivatives were synthesized via the reaction of thiophene carboxylic acid with various pyrazole amines. Here, 5-bromothiophene carboxylic acid (**2**) was reacted with 3-methyl-1-phenyl pyrazol-5-amine (**1**) via different methodologies (Scheme 1).

Protocol A: 5-bromothiophene carboxylic acid (0.35 g, 1.73 mmol), pyridine (17 mL), TiCl_4_ (0.56 mL, 5.19 mmol), 3-methyl-1-phenyl-1*H*-pyrazol-5-amine (0.3 g, 1.73 mmol), Protocol B: 5-bromothiophene carboxylic acid (3.58 g, 17.3 mmol), 3-methyl-1-phenyl-1*H*-pyrazol-5-amine (3.0 g, 17.3 mmol), *N,N*-dicyclohexylcarbodiimide (6.893 g, 29.41 mmol), 4(dimethyl amino)-pyridine (3.588 g, 29.41 mmol), DCM (150 mL) Protocol C: 3-methyl-1-phenyl-1*H*-pyrazol-5-amine (0.1 g, 0.577 mmol), 5-bromothiophene carboxylic acid (0.131 g, 0.634 mmol), 4-methylphenyl boronic acid (0.0008 g, 1 mol%, and 0.577 mmol), and toluene (10 mL). Protocol D: 3-methyl-1-phenyl-1*H*-pyrazol-5-amine (0.1 g, 0.577 mmol), 5-bromothiophene carboxylic acid (0.12 g, 0.577 mmol), Xylene (20 mL).

In Scheme 1, 5-bromo-*N*-(3-methyl-1-phenyl-pyrazol-5-yl)thiophene-2-carboxamide (3) was synthesized by the reaction of (1) and (2). In Protocol A, the 5-bromothiophene carboxylic acid (2) and 3-methyl-1-phenyl-1*H*-pyrazol-5-amine (1) was reacted in the presence of TiCl_4_. Here, pyridine was used as a base and a solvent. It was noted that the amide (3) showed the lowest (12%) yield (Table 1). Furthermore, in Protocol B, (1) and (2) were reacted in the presence of 4-(dimethyl-amino) pyridine (DMAP) as a catalyst, *N,N*-dicyclohexylcarbodiimide (DCC) as a coupling-agent, and dry DCM as a solvent. It was observed that a poor (8%) yield of the product (3) was obtained (Table 1). Moreover, in Protocol C, compounds (1) and (2) were refluxed at 90 °C by using 4-methylphenyl boronic acid and dry toluene to develop the amide-linkage. It was noted that the use of bulky and sterically hindered amine gave a poor (7%) yield (Table 1). Herein, Protocol D compounds (1) and (2) were refluxed at 90 °C by using xylene as a solvent, resulting in the development of an amide linkage, but product (**3**) gave the lowest yield (Table 1). Du and his co-workers reported that the protection on position 1 of the pyrazole ring gave a low yield compared to unprotected pyrazole, and the phenyl ring on pyrazole shows steric effects that make it less reactive [9]. Faria and co-workers observed that the lone pairs of NH_2_ also resonate in the pyrazole ring and many resonating structures of pyrazole are formed, which make it less reactive toward the electrophilic carbon of the acid and lowers the yield of the compounds [10].

**Scheme 2:**
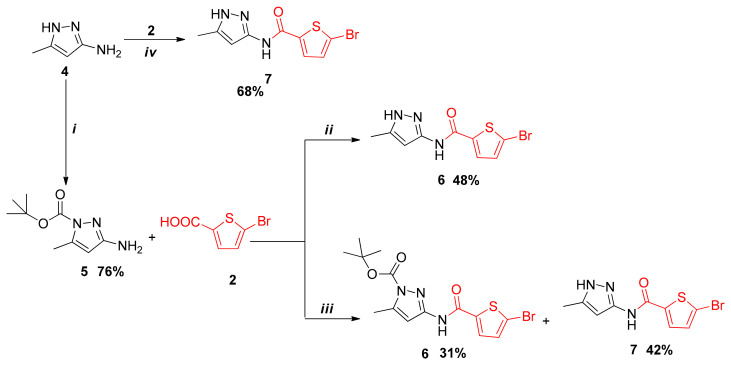
Synthesis after tert-butyl-3-(5-bromothiophene-2-carboxamido)-5-methyl-pyrazole-1-carboxylate.

Moreover, it was observed that when the nitrogen of pyrazole was substituted, it caused a hindrance and the products were obtained in low yields. Thus, for the direct synthesis of amide, we selected pyrazole, which has no protection on position 1. P. Rzepecki reported that the aromatic amine group was less reactive than the ring NH group [11]. The readily available hydrogen inhibits the synthesis of amide, necessitating initial protection of the pyrazole nitrogen [12,13]. Therefore, commercially available 5-methyl-1*H*-pyrazol-3-amine (**4**) was protected by using di-tert-butyl-dicarbonate for tert-butoxycarbonyl (BOC) protection (Scheme 2 (***i***)).

Furthermore, 5-bromo-*N*-(5-methyl-1*H*-pyrazol-3-yl)thiophene-2-carboxamide (**7**) was synthesized by two different methodologies (Scheme 2 (***ii, iii***)). The reaction was carried out between 5-bromothiophene carboxylic acid (**2**) and tert-butyl-3-amino-5-methylpyrazole-1-carboxylate (**5**), in the presence of TiCl_4_ and pyridine. It was also noted that the product (**6**) was not obtained; however, a 48 percent yield of an unprotected thiophene-based product containing pyrazole amide (**7**) was formed. In the literature survey, it was found that position-1 of pyrazole becomes deprotected due to the acidic workup, and the un-protected pyrazole amide was formed in the reaction [14]. Carmalt and his co-workers reported the complex formation of nitrogen-containing heterocycles with titanium [15]. It might be possible that the lesser yield of the compound (**7**) was obtained due to the complex formation. Furthermore, the compounds (**2**) and (**5**) were reacted in the presence of *N,N*-dicyclohexyl-carbodiimide, 4-(dimethylamino) pyridine, with dry DCM as a solvent, to form products (**6**) and (**7**). In this case, compound (**6**) gave 31% yield while that of (**7**) gave 42% yield (Scheme 2 (***iii***)). From the literature, it was observed that the basic workup also de-protects the BOC group. Subsequently, it was surprising that the protected amide (**6**) was also formed at about 31 percent.

Conditions: ***i:*** 5-methyl-1*H*-pyrazol-3-amine (1.0 g, 10.2965 mmol), di-tert-butyl-di-carbonate (3.3708 g, 15.444 mmol), Et_3_N (1.5628 g, 2.1526 mL, 15.444 mmol), 1,4-Dioxane (50 mL), Ethyl acetate (50 mL), saturated citric acid solution (50 mL). ***ii*:** reflux 2h, tert-butyl 3-amino-5-methyl-1*H*-pyrazole-1-carboxylate (0.1 g, 0.507 mL), 5-bromothiophene carboxylic acid (0.104 g, 0.507 mL), pyridine (10 mL) TiCl_4_ (0.1671 mL, 1.521 mmol). ***iii*:** tertbutyl-3-amino-5-methyl-pyrazole-1-carboxy-late (0.7 g, 3.55 mL), 5-bromothiophene carboxylic acid (0.735 g, 3.55 mmol), *N,N*-di-cyclo-hexyl-carbo-di-imide (1.414 g, 6.035 mmol), 4(dimethyl amino)-pyridine (0.736 g, 6.035 mmol), DCM (75 mL) ***iv:*** 5-methyl-1*H*-pyrazol-3-amine (1 g, 10.296 mmol), 5-bromothiophene carboxylic acid (2.13 g, 10.29 mmol), *N,N*-dicyclohexyl-carbodiimide (4.100 g, 17.493 mmol), 4 (dimethylamino)-pyridine (2.137 g, 17.493 mmol), DCM (150 mL).

Furthermore, Anna Kusakiewicz-Dawid reported amide formation without the protection of pyrazole by using the condensing agent DCC at room temperature [16]. Therefore, DCC and DMAP were used for direct amidation, without any protection. Then, 5-bromo-*N*-(5-methyl-1*H*-pyrazol-3-yl)thiophene-2-carboxamide (**7**) was obtained via the reaction of (**2**) and (**4**), without any protection, in the presence of DCC and DMAP (Scheme 2) (**iv**). It was observed that, during the basic workup, the amide was less soluble in the organic solvent and gave a lower yield, while a good (68%) yield of (**7**) was obtained by a simple filtration process.

**Scheme 3:**
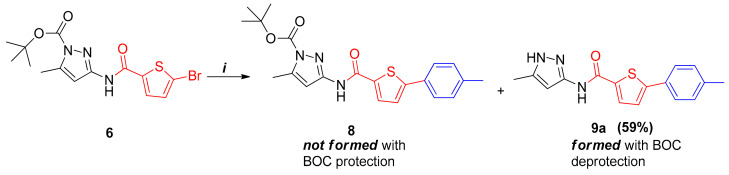
Arylation of tertbutyl-3-(5-bromothiophene-2-carboxamido)-5-methyl-pyrazole-1-carboxylate.

Christoph A. Fleckenstein and Herbert Plenio reported that the nitrogen-containing heterocycles have free amino groups, and the active centers were blocked due to nitrogen coordination. The nitrogen is also protected for the Suzuki–Miyaura cross-coupling. Thus, cross-coupling is less effective without protection [17]. However, the arylation of the protected amide tert-butyl 5-methyl-3-(5-(p-tolyl)thiophene-2-carboxamido)-1*H*-pyrazole-1-carboxylate (**6**) with methylphenyl boronic acid, with palladium (0) as a catalyst, was tried to promote Suzuki–Miyaura cross-coupling. It was observed that the deprotected product (**9a**) was obtained at 59% yield, while (**8**) was not formed (Scheme 3). Prashad and his co-workers reported the deprotection of the BOC group in the presence of an aqueous base [14]; the deprotection was also observed in the present study. It might be possible due to the use of a base (K_3_PO_4_) and distilled water in the Suzuki–Miyaura cross-coupling that de-protects the BOC group.

Conditions: ***i:*** bromothiophene-2-carboxamido)-5-methyl-1*H*-pyrazole-1-carboxylate (0.1 g, 0.349 mmol), 4-methylphenyl boronic acid (0.13 g, 0.768 mmol), tetrakis (triphenylphosphine)-Pd (0) (0.028 g, 2.443 mmol), potassium phosphate (0.349 g, 0.07 mmol), 1,4-dioxane: distilled water (4 mL:1 mL).

**Scheme 4:**
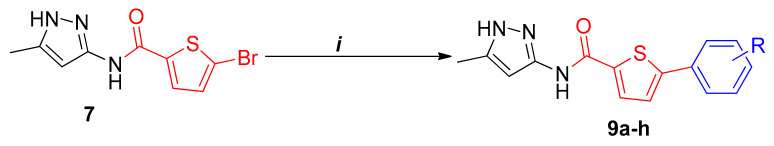
Arylation of 5-bromo-*N*-(5-methyl-1*H*-pyrazol-3-yl)thiophene-2-carboxamide.

**Figure 2:**
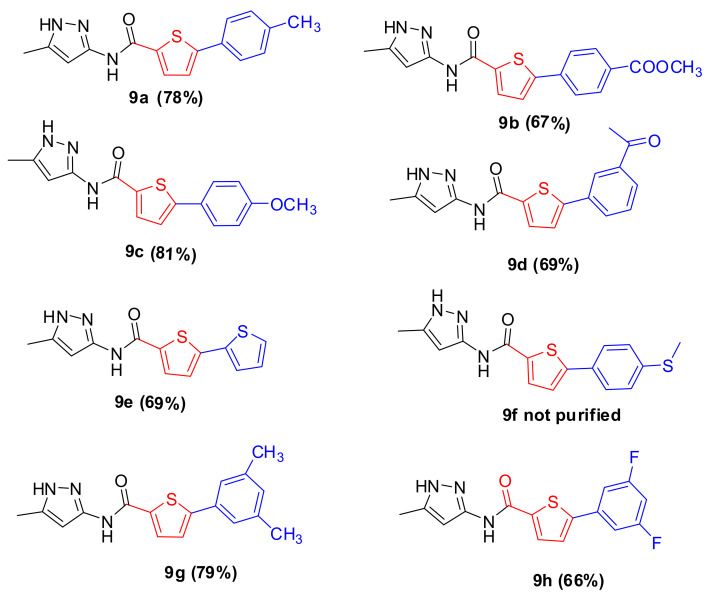
Products and percentage yields **9a**–**h**.

Moreover, Shaughnessy and co-workers reported the arylation of unprotected nitrogen-containing heterocycles via Suzuki–Miyaura cross-coupling in the presence of water-soluble Pd complexes, eliminating the need for protection–de-protection conditions [18,19]. An extension to cross-coupling was reported by chemists for higher yields but the excessive use of a catalyst and water-soluble ligands were required [20]. Interestingly, amide (**7**) was arylated via Suzuki–Miyaura cross-coupling with various aryl boronic acids. in the presence of 0.05 mol% palladium catalyst and 0.07 mol potassium phosphate in 1,4-dioxane (Scheme 4). Moderate to good yields of derivatives **9a**–**h** were obtained (Figure 2).

Conditions: (**i**) 5-bromo-*N*-(5-methyl-pyrazol-s3-yl)thiophene-2-carboxamide (1 eq.), hetero/aryl-boronic acid (1.1 eq.), tetrakis (triphenylphosphine)-palladium (0) (7 mol%), potassium phosphate (3 eq.), 1,4-dioxane: distilled water (4 mL:1 mL).

Furthermore, the compounds (**9c**) and (**9g**) gave very good yields of 81% and 79%, respectively, while compounds (**9a**–**9b**)**,** (**9d**–**9e**)**,** and (**9h**) gave moderate yields. It was observed that electronic-rich boronic acid gave a high yield, while electronic-poor boronic acids gave a moderate yield. Compound (**9f**) was not purified through column-chromatography (Figure 2).

### 2.2. Computational Studies

**Figure 3:**
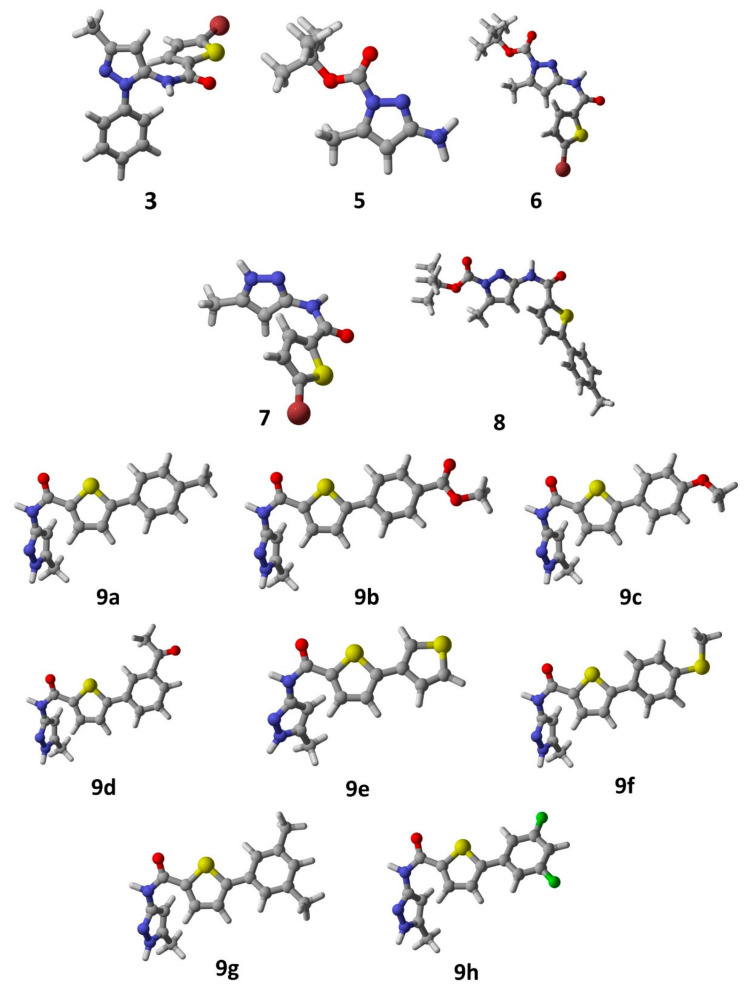
Optimized geometries of the minimum energy conformers of all the compounds under study (**3**, **5**–**8**, **9a**–**9h**) at the PBE0-D3BJ/def2-TZVP/SMD_DMSO_ level of theory. In 3D models, the grey color represents carbon, white represents hydrogen, yellow is for sulfur, green is for fluorine atoms, the red color is for oxygen, brown represents bromine, and the blue color shows nitrogen atoms.

All the pyrazole-based thiophene carboxamides (**3**, **5**–**8**, **9a**–**9h**, Figure 3) were modeled in three dimensions using a Gauss view and were then optimized at the PBE0-D3BJ/def2-TZVP/SMD_DMSO_ level of theory. This was followed by geometry optimization, then vibrational frequencies were computed and a relaxed potential energy scan was also performed, with the important dihedrals in consideration for all of the compounds to account for their conformers, using the same level of theory as described earlier. The resultant conformers of all the compounds were then subjected to unconstrained geometry optimizations, followed by the calculation of NMR and Boltzmann averaging of the NMR data of all the conformers. The Boltzmann-averaged NMR chemical shifts have been given in Table 2 for compound **9a,** while the rest of the compounds have been given in the supporting information; they show excellent agreement with the experimental chemical shifts. UV-Vis spectra have been computed at the TD-DFT/PBE0-D3BJ/def2-TZVP/SMD_DMSO_ level of theory. To investigate the nonlinear optical (NLO) responses of the compounds, dipole moments and hyperpolarizabilities have also been calculated according to the formulae given by Muhammad et al. [21], using the same level of theory as for the optimizations.

#### 2.2.1. Computation of NMR Data

Nuclear magnetic resonance (NMR) is a very important technique used by organic chemists to deduce/confirm the structures of synthesized compounds. DFT calculations of NMR chemical shifts can provide a very good NMR dataset to compare with the experimental one and will aid confidence in the NMR assignments. NMR calculations of all the modelled compounds have been conducted, on the same level of theory as the optimizations, and then compared with the experimental chemical shifts. Methanol has been used as a reference standard because of its better results, as previously described by Sarotti et al. [22]. Table 2 shows the comparison of the ^1^H-NMR data of compound **9a**. The comparison of all the other compounds has been given in the supporting information. It can be seen that the NMR computation approach has performed very well, with a mean absolute error (MAE) of only 0.24 ppm. Thus, the NMR data of the compounds that could not be obtained in a good yield to obtain their experimental NMR have been predicted with great confidence and can be used as a guide for the synthesis of these compounds in the future.

**Table 2:** Comparison of experimental and computed NMR data for compound **9a**.

Compound 9a				
Carbon No.	Carbon Type	^1^H-NMR (Experimental) δ, ppm	^1^H-NMR (Computed) δ, ppm	Δδ, ppm
2	C	-	-	-
3	CH	8.06	7.77	0.29
4	CH	7.50	7.74	0.24
5	C	-	-	-
1′N	NH	12.15	9.12	3.03
3′	C	-	-	-
4′	CH	6.36	6.86	0.50
5′	C	-	-	-
5′-Me	CH_3_	2.23	2.25	0.02
1″	C	-	-	-
2″	CH	7.62	8.14	0.52
3″	CH	7.26	7.66	0.40
4″	C	-	-	-
4″-Me	CH_3_	2.33	2.38	0.05
5″	CH	7.26	7.56	0.30
6″	CH	7.62	7.95	0.33
Mean Absolute Error (MAE) = 0.24 Root Mean Square Error (RMSE) = 0.65				

#### 2.2.2. Frontier Molecular Orbital (FMO) Analysis and Hyperpolarizability

**Figure 4:**
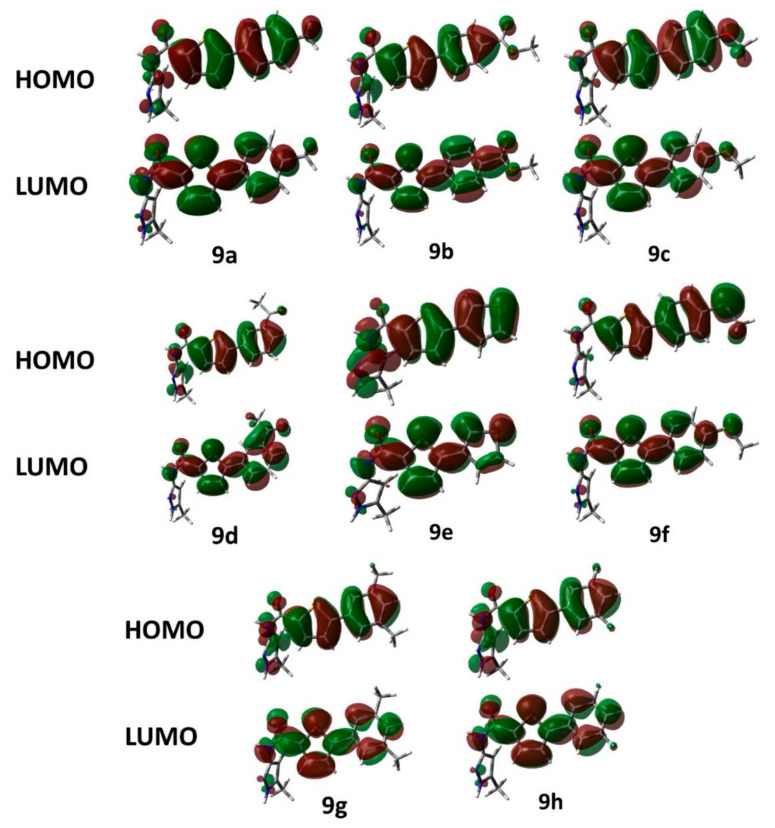
A presentation of the frontier orbitals of the molecules (**9a**–**9h**) calculated at the PBE0-D3BJ/def2-TZVP/SMD_DMSO_ level of theory.

FMO calculations provide useful information about reactivity and other properties of a chemical compound that can be extracted from the energies of the highest occupied molecular orbital (HOMO) and the lowest unoccupied molecular orbital (LUMO) [23]. Furthermore, the gap between the filled HOMOs and empty LUMOs, as well as their energy gap, are crucial quantum chemical factors for determining the chemical stability and reactivity of molecules. The compound with the highest energy gap between HOMO and LUMO is estimated to be the most stable and the least reactive, while the molecule with the lowest energy gap is unstable and the most reactive undergo aromatic substitution reactions readily. Comparison of the surface plots of FMOs with the experimental data provides important information about the ability of the computational method to describe the chemical reactivity of the molecules. Figure 4 contains the surface plots of the FMOs for compounds **9a**–**9h**. The energies of HOMO, LUMO, and their gap (ΔE), along with their hyperpolarizability (*β*) values, have been presented in Table 3 below.

The general reactivity of compounds (**9a**–**9h**) is similar because of their same skeleton, demonstrating different groups on them that can be seen from a narrow range of ΔE values of the compounds under study (4.30 eV to 4.54 eV). Compounds **9c** and **9h** have similar ΔE values, coincidentally, which makes both of them the most reactive in the series. One (**9h**) has two fluoride groups that are electron-withdrawing, and the other (**9c**) has a methoxy group on the aromatic ring that is electron-donating in nature. The highest ΔE value is of **9d** and is 4.54 eV; this can be attributed to the highly deactivating (acetyl) group on the aromatic ring, which makes it the least reactive in the series. The dispersion of iso-density has been shown in Figure 4.

Hyperpolarizability (*β*) has been computed for all the compounds to gain insight as to whether the compounds have a promising non-linear optical (NLO) response. Conjugated organic molecules with electron-donating and accepting moieties exhibit different values of the first hyperpolarizability. Moreover, a stronger donor and acceptor substituent on the aromatic ring improves charge transfer between the substituent and the ring in the electronic ground state, increases the oscillator strength, and significantly enhances the excitation-induced change of the dipole moment.

To examine the direction of change transfer, the computed values of the dipole moment (µ), and hyperpolarizability (*β*_tot_, *β*_vec_) have been listed in Table 4. The ratio of *β*_vec_ and *β*_total_ provides significant information regarding the direction of charge transfer within the compounds and is represented as: *β*_vec_/*β*_tot_ = cos θ, where θ represents the angle between the vector formed by the *β*_vec_ components and the dipole moment vector. In general, if this ratio approaches unity (*β*_tot_ and *β*_vec_ having equal amplitudes), it indicates that the charge transfer within the molecular system is unidirectional and parallel to the molecular dipole moment, such that one of the components contributes to almost all of the NLO response, thus approximating the *β*_vec_ and *β*_tot_ values.

Only compound **9f** showed a very good *β* value, which may be attributed to the two sulfur atoms in the vicinity of the aromatic ring. One -SCH_3_ group governs the electrons’ push and pull mechanism through the aromatic ring, due to its strongly activating nature, thus causing the compound to behave as a good NLO material. Compound **9c** has a similar structure but demonstrates a methoxy group instead of the -SCH_3_ group; its *β* value is the second-highest among the series.

#### 2.2.3. Molecular Electrostatic Potential (MESP)

**Figure 5:**
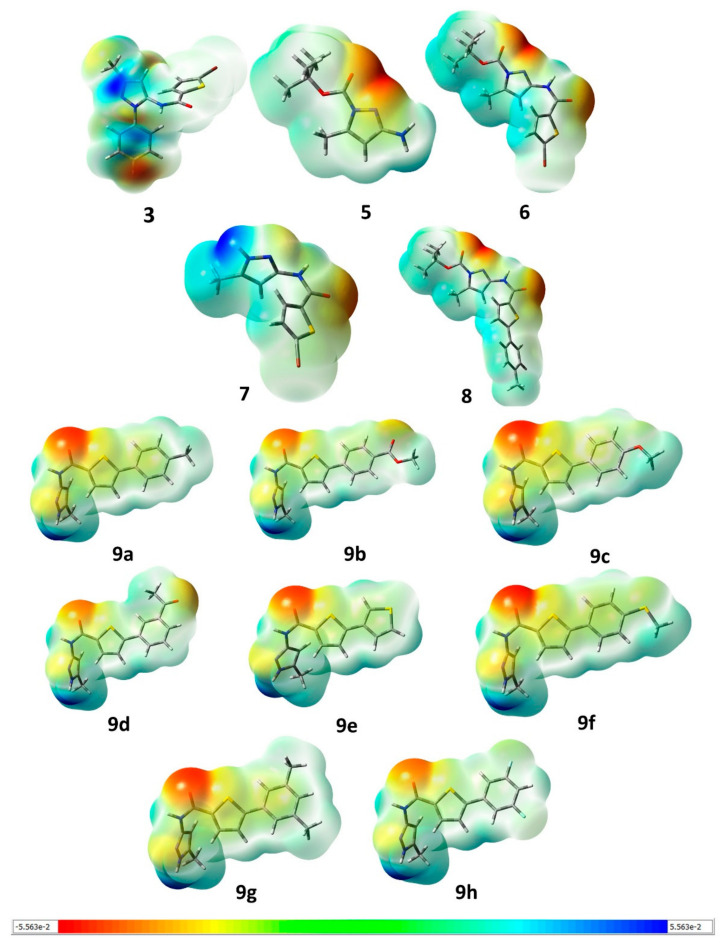
A presentation of all the molecular electrostatic potentials of the molecules (**3**, **5**–**7**, **8a**–**8b**, **9a**–**9h**), calculated at the PBE0-D3BJ/def2-TZVP/SMD_DMSO_ level of theory. The scale below shows the color-coding. The values in the scale are in hartrees.

Molecular electrostatic potential (MESP) can be plotted as a valuable tool to visualize the size of the molecule, its distribution of charges, and its overall shape. Different colors are used to show different charges and the electrons’ density. The red color is used to show the electron-rich sites, whereas the blue shade represents the electron-poor positions of the molecule. Figure 5 shows the MESP plots of the compounds under study. These plots can be helpful to visualize the overall geometry and space of the molecules.

#### 2.2.4. UV-Vis Spectral Analysis

DFT computations were used to examine the UV-Vis spectra of the compounds. Two main molecular orbitals (HOMO and LUMO) were studied for the compounds by drawing surfaces for the frontier orbitals to understand the bonding pattern. Calculations of molecular orbital geometry demonstrate that the absorption maxima represent the electronic transitions between frontier orbitals, such as translation from HOMO to LUMO. Furthermore, the charge density of the compounds in their HOMOs is localized mainly on the pyrazole ring, whereas LUMO is characterized by a charge distribution on the substituted thiophene ring, as shown in Figures S9–S16 (Supplementary Materials).

Calculated UV-Vis spectra have also been presented in the supporting information (Figures S9–S16). Two prominent absorption bands can easily be seen in the spectra of most of the compounds, each of which corresponds to a number of overlapping transitions. The blue lines indicate the frequencies of the individual excitations, and their height is proportional to the oscillator strength. The difference in the absorption patterns of the compounds near 250 nm to 300 nm can also be examined, which is due to the presence of distinct substituents on the thiophene ring. Subsequently, substitution significantly affects the absorption pattern of the compounds. For example, compounds including 9e, 9f, and 9h have shown a shift in absorption to a longer wavelength (redshift) around 330 nm. Furthermore, compounds 9c and 9e exhibit blue shifts at about 225.39 nm and 223.57 nm, respectively.

#### 2.2.5. Conceptual DFT Reactivity Descriptors

The DFT computations are also a source of other important information, including some important chemical reactivity descriptors, as shown in Table 5. These chemical reactivity descriptors have been calculated from the energies of HOMO and LUMO by the equations previously described by us in [24].

The predicted reactivity of the compounds **9c**, **9g**, and **9h** are confirmed by the reactivity descriptors, as shown in Table 5. The higher reactivity of compounds **9c** and **9h** can be related to their lowest ionization potentials and electron affinities, these being among the highest for **9d,** which was predicted to be the most stable in the series.

## 3. Materials and Methods

### 3.1. General Information

The rota-evaporator (Buchi, Baden-Württemberg, Germany) was used to distill all the solvents used in reactions. In the spectroscopic analysis, a 600 MHz Bruker NMR spectrometer (Billerica, MA, USA) was employed to check the structures of the synthesized compounds in the presence of a solvent, i.e., dimethyl sulfoxide-d_6_ (DMSO-d_6_). The moisture- and air-sensitive reagents were prevented by standard techniques. All the air and moisture-sensitive reactions were conducted under an inert atmosphere, particularly in the presence of argon. Moreover, Sigma Aldrich (Burlington, MA, USA) and Alfa-Aesar (Ward Hill, MA, USA) provided all the chemicals that were utilized in this experiment.

### 3.2. Procedure for the Synthesis of Compound (***3***)

*Protocol A:* The 5-bromothiophene carboxylic acid (0.35 g, 1.73 mmol) and pyridine (17 mL) was put in a Schlenk flask and stirred for 10–15 min at room temperature. After 10 min, TiCl_4_ (0.56 mL, 5.19 mmol) was added to the reaction mixture. After that, 3-methyl-1-phenylpyrazol-5-amine (0.3 g, 1.73 mmol) was put in the Schlenk flask, and stirring was continued for 2 h at 80 °C. Thin-layer chromatography (TLC) (Merck, Kenilworth, NJ, USA) was used to check the completion of the reaction. After 2 h, toluene was added to the reaction mixture and dried over a vacuum pump. After drying the reaction mixture, the workup of the reaction mixture was performed by adding 1N HCl solution, a saturated solution of sodium carbonate (Na_2_CO_3_), and dichloromethane (DCM) in the separating funnel. Then, the DCM (lower) layer was collected, and the solvent was evaporated on a rotary evaporator. Column chromatography was used to separate the components of the reaction mixture, and the product was analyzed using various techniques like ^1^H-NMR, and ^13^C-NMR [25,26].

*Protocol B:* 5-bromothiophene carboxylic acid (3.58 g, 17.3 mmol) and 3-methyl-1-phenyl-1*H*-pyrazol-5-amine (3.0 g, 17.3 mmol) were dissolved in 150 mL DCM, followed by the addition of 4-dimethylamino-pyridine (DMAP) (3.588 g, 29.41 mmol). The temperature of the reaction mixture was dropped to 0 °C in an isotherm with continuous stirring. When the reaction mixture attained a temperature of 0 °C, then *N,N*-dicyclohexylcarbodiimide (DCC) (6.893 g, 29.41 mmol) was added to an inert atmosphere. After that, the reaction mixture was taken up to room temperature by removing the isotherm. Then, the reaction mixture was allowed to stir for 18–52 h at room temperature. TLC was used to check the completion of the reaction. The reaction mixture, DCM, saturated solution of Na_2_CO_3_, and saturated brine solution were put in the reaction mixture in a separating funnel after completion of the reaction. Then, the DCM (lower) layer was collected, and the solvent was evaporated on a rotary evaporator. Column chromatography (CC) was used to separate the products from the reaction mixture. The synthesized product was verified using different techniques like ^1^H-NMR,^13^C-NMR, and elemental analysis [27].

*Protocol C:* 3-methyl-1-phenyl-1*H*-pyrazol-5-amine (0.1 g, 0.577 mmol), 5-bromothiophene carboxylic acid (0.131 g, 0.634 mmol), 4-methyl phenyl-boronic acid (0.0008 g, 1 mol%, and 0.577 mmol) and toluene (10 mL) were put in a Schlenk flask. The reaction mixture was put on the reflux for 42 h. TLC was used to monitor the completion of the reaction. A rotary evaporator was used after the completion of the reaction for evaporating the solvent. The components of the reaction mixture were separated by CC. The synthesized product was verified using various techniques like ^1^H-NMR,^13^C-NMR, and elemental analysis [28].

*Protocol D:* 3-methyl-1-phenyl-1*H*-pyrazol-5-amine (0.1 g, 0.577 mmol, 5-bromothiophene carboxylic acid (0.12 g, 0.577 mmol) and xylene (20 mL) were added to the Schlenk flask. The flask was put on the reflux for 48 h. The reaction was monitored by TLC. A rotary evaporator was used after the reaction completion for evaporating the solvent. CC was employed to separate the components of the reaction mixture. The synthesized product was verified using various techniques like ^1^H-NMR, ^13^C-NMR, and elemental analysis [29].

### 3.3. Procedure for the Synthesis of Tert-butyl 3-amino-5-methyl-1H-pyrazole-1-carboxylate (***5***)

***(i)*** 5-methyl-1*H*-pyrazol-3-amine (1.0 g, 10.2965 mmol), di-tert-butyl-tri-carbonate (3.3708 g, 15.444 mmol), and Et_3_N (1.5628 g, 2.1526 mL, 15.444 mmol) were dissolved in 1,4-dioxane (50 mL) in a Schlenk flask, followed by refluxing the reaction mixture for 3 h. TLC was used to check the reaction completion. A rotary evaporator was used after the reaction was completed, to evaporate the solvent. Ethyl acetate (50 mL) and a saturated citric acid solution (50 mL) were added to the dried reaction mixture, then the ethyl acetate layer was collected and subjected to CC, to separate the components of the reaction mixture. The synthesized product was verified using various techniques like ^1^H-NMR, ^13^C-NMR, and elemental analysis [16].

### 3.4. Synthesis of Tertbutyl-3-(5-bromothiophene-2-carboxamido)-5-methyl-pyrazole-1-carboxylate (***6***)

(***ii***) 5-bromothiophene carboxylic acid (0.104 g, 0.507 mL) and pyridine (10 mL) were put in a Schlenk flask and stirred for 15 min at room temperature. After 10 min, TiCl_4_ (0.1671 mL, 1.521 mmol) was added to the reaction mixture. After that, tertbutyl-3-amino-5-methylpyrazole-1-carboxylate (0.1 g, 0.507 mL) was added to the reaction medium, followed by heating the reaction mixture at 80 °C for 2 h. The toluene was added to it, then the pyridine and toluene were evaporated by a rotary evaporator after the reaction was complete. Then, the dried reaction mixture, 1N HCl solution, DCM, and saturated solution of sodium carbonate (Na_2_CO_3_) were added to the separating funnel and DCM (lower) layer was collected and dried on a rotary evaporator. The components of the reaction mixture were separated by CC. The synthesized product was verified using various techniques like ^1^H-NMR, ^13^C-NMR, and elemental analysis [25].

(***iii***) 5-bromothiophene carboxylic acid (0.735g, 3.55mmol), tertbutyl-3-amino-5-methyl-pyrazole-1-carboxylate (0.7 g, 3.55 mL) were put in 75 mL DCM in a Schlenk flask, followed by the addition of DMAP (0.736 g, 6.035 mmol) to the reaction mixture. The Schlenk flask was cooled in an isotherm at 0 °C. When the reaction mixture attained a temperature of 0 °C, DCC (1.414 g, 6.035 mmol) was added. The reaction proceeded in an inert atmosphere. Then, the reaction mixture was taken back to room temperature and continued to be stirred for 18–52 h. TLC was used to check the reaction completion. A rotary evaporator was used after the reaction was complete, for evaporating the solvent. The dried reaction mixture, DCM, saturated brine solution, and Na_2_CO_3_ were added together in a separating funnel after the reaction was complete, followed by collecting the DCM (lower) layer and evaporating the solvent on a rotary evaporator. The components of the reaction mixture were separated by CC. The product was verified using different techniques like ^1^H-NMR, ^13^C-NMR, and elemental analysis [27].

### 3.5. Procedure for the Synthesis of 5-Bromo-N-(5-methyl-1H-pyrazol-3-yl)thiophene-2-carboxamide (***7***)

The 5-bromothiophene carboxylic acid (0.735 g, 3.55 mmol), 5-methyl-1*H*-pyrazol-3-amine and DMAP (0.736 g, 6.035 mmol) were put in 75 mL DCM in a Schlenk flask. The reaction mixture was cooled to 0 °C in an isotherm. When the reaction mixture attained a temperature of 0 °C, DCC (1.414 g, 6.035 mmol) was added. The reaction was conducted in an argon environment. After that, the reaction mixture was taken back to normal temperature by removing the isotherm, and the reaction mixture was stirred for 18–52 h. TLC was used to check the reaction was complete. A rotary evaporator was used after the reaction was complete, for evaporating the solvent. The dried reaction mixture was filtered and washed with ethyl acetate, and a white powder was collected from the filter paper. CC was used to separate the components of the reaction mixture. The synthesized product was analyzed using ^1^H-NMR, ^13^C-NMR, and elemental analysis [27].

### 3.6. Synthesis of N-(5-Methyl-1H-pyrazol-3-yl)-5-(p-tolyl)thiophene-2-carboxamide (***8b***)

The tert-butyl 3-(5-bromothiophene-2-carboxamido)-5-methyl-1*H*-pyrazole-1-carboxylate (0.1 g, 0.349 mmol), 1, 4-dioxane (4 mL) and tetrakis-(triphenylphosphine)-palladium (0) (0.028 g, 2.443 mmol) were placed in a Schlenk tube under an inert environment for half an hour, with stirring at room temperature. After half an hour, 4-methylphenyl boronic acid (0.13 g, 0.768 mmol) and base K_3_PO_4_ (0.349 g, 0.07 mmol) were added to the reaction mixture. Then, distilled water (1 mL) was added to the reaction mixture and was kept at 85–95 °C for 18 h under an inert environment. TLC was used to check the reaction was complete. After completion, ethyl acetate was used to wash and filter the reaction mixture. A rotary evaporator was used to evaporate the solvent. The product from the reaction mixture was purified by CC. The synthesized product was analyzed by ^1^H-NMR, ^13^C-NMR, and elemental analysis [30,31].

### 3.7. Arylation of 5-Bromo-N-(5-methyl-1H-pyrazol-3-yl)Thiophene-2-carboxamide (***9a***–***h***)

The 5-bromo-*N*-(5-methyl-pyrazol-3-yl)thiophene-2-carboxamide (1 eq.), 1,4-dioxane (4 mL), and tetrakis-(triphenylphosphine)-palladium (0) (7mol%) were placed together in a Schlenk tube under an inert environment and stirred for half an hour at room temperature. After half an hour, hetero/aryl boronic (1.1 eq.) and the base K_3_PO_4_ (3 eq.) were added to the reaction mixture. After that, distilled water (1 mL) was added to the reaction mixture, and it was kept at 85–95 °C for 18 h under an inert environment. TLC was used to check the reaction was complete. After completion, the reaction mixture was filtered and washed with ethyl acetate. A rotary evaporator was used to evaporate the solvent. The product from the reaction mixture was purified by CC. The synthesized product was analyzed using ^1^H-NMR, ^13^C-NMR, and elemental analysis [32,33].

### 3.8. Computational Method

The use of computational chemistry tools to augment confidence in experimental techniques has seen a rise in the past decade, due to the widespread availability of powerful computers. This study also benefitted from computational tools; the software used was Gaussian 09 [34] (Gaussian, Inc., Pittsburgh, PA, USA). The functional used was PBE0 [35,36], which is a hybrid-density functional, along with a triple ζ quality basis set def2-TZVP [37] for all the calculations. Grimme’s empirical dispersion correction, with Becke–Johnston damping (D3BJ) [38,39,40], has been added to account for dispersion interactions during the optimizations. The polarizable continuum model (PCM) [41,42,43,44,45,46,47] has been used, accompanying the SMD parameter set by Truhlar [48], for the solvent effects, available in Gaussian 09. DMSO has been used as a solvent in all the calculations. Frequency calculations at the same level of theory have been employed as a tool to confirm the optimized structures to be true minima. True minima are structures without imaginary frequencies.

### 3.9. Characterization

**5-bromo-*N*-(3-methyl-1-phenylpyrazol-5-yl)thiophene-2-carboxamide (3).**^1^H-NMR (600 MHz, DMSO): 10.37 (s, 1H), 7.68(d, *J = 3.6Hz*, 1H), 7.51(m, 2H), 7.46(m, 2H), 7.35 (m, 1H), 7.32(s, 1H), 6.27 (s, 1H), 2.25 (s, 3H); ^13^CNMR (126 MHz, DMSO): δ 161.4, 149.3, 148.2, 140.1, 139.5, 138.2, 132.1, 129.4, 124.3, 123.6, 123.3, 91.1, 13.2. Analysis calculated for C_15_H_12_BrN_3_OS: C,49.76; H,3.33; N,11.62 Found: C,49.78; H,3.36; N,11.59.

**Tert-butyl-3-amino-5-methylpyrazole-1-carboxylate (5).**^1^H-NMR (600 MHz, DMSO): 6.52 (s, 1H), 6.01 (s, 2H), 2.46 (s, 3H), 1.37 (s, 9H); ^13^CNMR (126 MHz, DMSO): δ 152.1, 144.5, 139.3, 92.1, 88.7, 50.9, 31.1, 29.8, 14.5. Analysis calculated for C_11_H_19_N_3_: C, 68.35; H, 9.91; N, 21.74 Found: C, 68.32; H, 9.89; N, 21.72.

**Tert-butyl 3-(5-bromothiophene-2-carboxamido)-5-methylpyrazole-1-carboxylate (6).**^1^H-NMR (600 MHz, DMSO): 11.88 (s,1H), 7.91 (d, *J* = 6Hz,1H), 7.31 (d, *J* = 6Hz, 1H), 6.32(s,1H), 2.22 (s,3H), 1.94 (s,9H); ^13^C NMR (126 MHz, DMSO): δ 161.9, 149.3, 143.3, 140.2, 139.5, 138.7, 132.1, 124.3, 88.3, 83.5, 28.6, 13.2. Analysis calculated for C_14_H_16_BrN_3_O_3_S: C, 43.53; H, 4.18; N, 10.88 Found: C, 43.55; H, 4.19; N, 10.86.

**5-bromo-*N*-(5-methyl-1*H*-pyrazol-3-yl)thiophene-2-carboxamide (7).**^1^H-NMR (600 MHz, DMSO): 12.15 (s, 1H), 10.86 (s, 1H), 7.90(d, *J* = 6 Hz,1H), 7.30 (d, *J* = 6 Hz,1H), 6.32(s, 1H), 2.21 (s, 3H); ^13^C NMR (126 MHz, DMSO): δ 161.9, 148.7, 140.3, 138.7, 138.1, 132.2, 124.1, 98.4, 13.2. Analysis calculated for C_9_H_8_BrN_3_OS: C,37.78; H,2.82; N,14.68 Found: C,37.79; H,2.83; N,14.65.

***N*-(5-methyl-pyrazol-3-yl)-5-p-tolyl-thiophene-2-carboxamide (9a).**^1^H-NMR (600 MHz, DMSO): 12.12 (s, 1H), 10.76 (s, 1H), 8.06 (d, *J* = 4.2Hz, 2H), 7.62 (d, *J* = 7.8Hz, 2H), 7.50 (d, *J* = 4.2Hz, 2H), 7.26 (d, *J*= 7.8Hz, 2H), 6.36 (s, 1H), 2.37 (s, 3H), 2.23 (s, 3H); ^13^C NMR (126 MHz, DMSO): δ 161.9, 148.7, 148.1, 138.3, 138.1, 137.3, 131.4, 130.5, 129.7, 129.3, 125.7, 98.1, 21.4, 13.2. Analysis calculated for C_16_H_15_N_3_OS: C, 64.62; H, 5.08; N, 14.13 Found: C, 64.65; H, 5.11; N, 14.11

**Methyl-4-(5-(1*H*-pyrazol-3-ylcarbamoyl)thiophene-2-yl)-benzoate (9b).**^1^H-NMR (600 MHz, DMSO): 12.15 (s, 1H), 10.87 (s, 1H), 8.08 (d, *J* = 6Hz, 1H), 8.02 (d, *J* = 6Hz, 2H), 7.89 (d, *J* = 12Hz, 2H), 7.72 (d, *J* = 6Hz, 1H), 6.37 (s, 1H), 3.88 (s, 3H), 2.23 (s, 3H); ^13^C NMR (126 MHz, DMSO): δ 165.7, 161.9, 148.1, 138.3, 138.1, 137.3, 135.2, 132.1, 130.3, 129.6, 127.3, 127.3, 91.7, 51.2. Analysis calculated for C_17_H_15_N_3_O_3_S: C, 59.81; H, 4.43; N, 12.31 Found: C, 59.82; H, 4.45; N, 12.29.

**5-(4-methoxyphenyl)-*N*-(5-methylpyrazol-3-yl)thiophene-2-carboxamide (9c).**^1^H-NMR (600 MHz, DMSO): 12.12 (s, 1H), 10.76 (s, 1H), 8.06 (d, *J* = 4.2Hz, 1H), 7.62 (d, *J* = 7.8Hz, 2H), 7.50 (d, *J* = 4.2Hz, 1H), 7.26 (d, *J* = 7.8Hz, 2H), 6.36 (s, 1H), 2.6 (s, 3H), 2.23 (s, 3H); ^13^C NMR (126 MHz, DMSO): δ 161.9, 160.4, 148.9, 148.3, 138.3, 138.1, 137.4, 129.5, 128.3, 126.1, 114.7, 98.5, 55.9, 13.2. Analysis calculated for C_16_H_15_N_3_O_2_S: C, 61.32; H, 4.82; N, 13.41 Found: C, 61.34; H, 4.83; N, 13.38.

**5-(4-acetylphenyl)-*N*-(5-methyl-1*H*-pyrazol-3-yl)thiophene-2-carboxamide (9d).**^1^H-NMR (600 MHz, DMSO): 12.14 (s, 1H), 10.84 (s, 1H), 8.40 (s, 1H), 7.98 (m, 4H), 7.70 (d, *J* = 6Hz, 1H), 6.37 (s, 1H), 2.66 (s, 6H), 2.23 (s, 3H); ^13^C NMR (126 MHz, DMSO): δ 196.8, 161.9, 148.7, 148.1, 138.4, 138.1, 137.4, 133.5, 130.9, 129.6, 129.3, 128.7, 126.2, 98.5, 26.7, 13.2. Analysis calculated for C_17_H_15_N_3_O_2_S: C, 62.75; H, 4.65; N, 12.91 Found: C, 62.77; H, 4.67; N, 12.88.

***N*-(5-methyl-1*H*-pyrazol-3-yl)-2, 2′-bithiophene-5-carboxamide (9e).**^1^H-NMR (600 MHz, DMSO): 12.12 (s, 1H), 10.75 (s, 1H), 8.04 (d, *J* = 4.2Hz, 1H), 7.89 (s, 1H), 7.67 (dd, *J* = 6Hz,6Hz, 1H), 7.49 (dd, *J* = 6Hz,6Hz, 1H), 7.43 (d, *J* = 6.0Hz, 1H), 6.35 (s, 1H), 2.22 (s, 3H); ^13^C NMR (126 MHz, DMSO): δ 161.9, 148.7, 143.5, 138.4, 138.1, 137.4, 136.8, 133.5, 128.3, 124.5, 124.2, 98.6, 13.2. Analysis calculated for C_13_H_11_N_3_OS_2_: C, 53.96; H, 3.83; N, 14.52 Found: C, 53.99; H, 3.84; N, 14.51.

**5-(3,5-dimethylphenyl)-*N*-(5-methyl-1*H*-pyrazol-3-yl)thiophene-2-carboxamide (9g).**^1^H-NMR (600 MHz, DMSO): 12.12 (s, 1H), 10.76 (s, 1H), 8.05 (s, 1H), 2.23(s, 3H), 7.50 (d, *J* = 6Hz, 1H), 7.34 (s, 2H), 7.02 (s, 1H), 6.36 (s, 1H), 2.32 (s, 3H); ^13^C NMR (126 MHz, DMSO): δ 161.9, 148.7, 148.3, 138.8, 138.4, 138.1, 137.3, 133.6, 131.1, 129.3, 127.5, 98.5, 21.7, 13.2. Analysis calculated for C_17_H_17_N_3_OS: C, 65.57; H, 5.50; N, 13.49 Found: C, 65.58; H, 5.52; N, 13.47.

**5-(3,5-difluorophenyl)-*N*-(5-methyl-1*H*-pyrazol-3-yl)thiophene-2-carboxamide (9h).**^1^H-NMR (600 MHz, DMSO):12.15 (s, 1H), 10.88 (s, 1H),8.09 (s, 1H), 7.37 (m, 2H), 7.26 (m,1H),7.11 (m, 1H),6.98 (d, *J* = 12, 1H),6.36 (s, 1H), 2.23 (s, 3H); ^13^C NMR (126 MHz, DMSO): δ 165.2, 161.7, 165.1, 148.4, 148.9, 138.4, 138.1, 137.3, 136.7, 129.5, 111.7, 104.9, 98.5, 13.2. Analysis calculated for C_15_H_11_F_2_N_3_OS: C, 56.42; H, 3.47; N, 13.16 Found: C, 56.44; H, 3.48; N, 13.15.

## 4. Conclusions

In this paper, 5-bromo-*N*-(3-methyl-1-phenylpyrazol-5-yl)thiophene-2-carboxamide (**3**) was synthesized via different methodologies. A poor yield of (**3**) was observed from all the methods utilized, due to the presence of the phenyl ring on the pyrazole moiety. The substituted pyrazole ring gave a low yield, compared to un-substituted pyrazole, and the phenyl ring on pyrazole showed steric effects that made it less reactive. The un-substituted pyrazole-based thiophene amide was synthesized and arylated via the Suzuki–Miyaura cross-coupling reaction, giving a moderate to good yield. DFT calculations on the synthesized compounds have led to an understanding of their geometry and other physical properties, like NLO properties, NMR, and other chemical reactivity descriptors including the electron affinity, ionization potential, electronic chemical potential, chemical hardness, and electrophilicity index. NMR calculations have shown a very good agreement with the experimental NMR data that was available. This also led to confidently predicted NMR data of the compounds that could not be obtained in a good yield, to perform their NMR spectroscopy. One compound (**9f**) also showed a very good NLO response, which means that it can act as a good NLO material. FMO analysis and the reactivity descriptors predicted compounds (**9c**) and (**9h**) to be the most reactive chemically, while (**9d**) has been predicted to be the most stable among the studied series of compounds.

## Figures and Tables

**Table 1:** The percentage yield of (**3**) by different methodologies.

Entry	Catalyst	Solvent	Base	% Yield
3	TiCl_4_	Pyridine	Pyridine	12
	DCC/DMAP	DCM	-	8
	4-methylphenyl boronic acid	Toluene	-	7
	-	Xylene	-	9

**Table 3:** The energies of HOMO, LUMO, and the gap between HOMO and LUMO. All the HOMO and LUMO energies are given in eV. Hyperpolarizability (*β*) values are given in hartrees.

Compound	E*_HOMO_*	E*_LUMO_*	HOMO-LUMO Gap	Hyperpolarizability
**3**	−6.67	−1.68	4.99	1597.42
**5**	−6.01	−0.35	5.66	1568.38
**6**	−6.70	−1.67	5.03	1570.87
**7**	−6.76	−1.55	5.21	882.79
**8a**	−6.20	−1.79	4.41	4477.25
**8b**	−6.16	−1.72	4.44	3174.72
**9a**	−6.16	−1.71	4.45	3181.71
**9b**	−6.44	−2.10	4.33	3785.18
**9c**	−5.92	−1.62	4.30	6825.00
**9d**	−6.39	−1.86	4.54	1144.23
**9e**	−6.14	−1.75	4.40	2517.82
**9f**	−5.79	−1.74	4.45	10,221.04
**9g**	−6.23	−1.70	4.33	1876.18
**9h**	−6.44	−1.93	4.30	799.16

**Table 4:** Dipole moments (µ), and hyperpolarizabilities (β_vec_, β_tot_) (calculated at PBE0/def2TZVP_DMSO_) of the compounds under study.

Compounds	µ (Debye)	β_tot_ (a.u.)	β_vec_ (a.u.)	β_vec_/β_tot_
**3**	3.561	176.66	−80.6	−0.456
**5**	3.776	135.50	−15.6	−0.115
**6**	7.433	352.12	113.7	0.322
**7**	9.127	241.55	130.4	0.539
**8**	9.265	376.69	227.2	0.603
**9a**	7.416	206.53	188.2	0.911
**9b**	7.396	233.41	229.0	0.981
**9c**	5.574	229.21	111.4	0.486
**9d**	9.795	612.59	449.1	0.733
**9e**	8.316	424.17	210.0	0.495
**9f**	10.543	452.47	347.5	0.853
**9g**	8.734	237.68	231.2	0.972
**9h**	9.387	259.34	259.5	1.001

**Table 5:** Ionization potential (*I*), electron affinity (*A*), chemical hardness (*η*), electronic chemical potential (*µ*), and electrophilicity index (*ω*) of the compounds under study.

Compound	Ionization Potential, *I* (eV)	Electron Affinity, *A* (eV)	Chemical Hardness, *ƞ* (eV)	Electronic Chemical Potential, *μ* (eV)	Electrophilicity Index, *ω* (eV)
**3**	6.67	1.68	−2.49	4.17	−3.49
**5**	6.01	0.35	−2.83	3.18	−1.79
**6**	6.70	1.67	−2.52	4.18	−3.48
**7**	6.76	1.55	−2.61	4.16	−3.32
**8a**	6.20	1.79	−2.20	4.00	−3.62
**8b**	6.16	1.72	−2.22	3.94	−3.49
**9a**	6.16	1.71	−2.22	3.94	−3.48
**9b**	6.44	2.10	−2.17	4.27	−4.21
**9c**	5.92	1.62	−2.15	3.77	−3.31
**9d**	6.39	1.86	−2.27	4.13	−3.75
**9e**	6.14	1.75	−2.20	3.94	−3.54
**9f**	6.16	1.71	−2.22	3.94	−3.48
**9g**	6.44	2.10	−2.17	4.27	−4.21
**9h**	5.92	1.62	−2.15	3.77	−3.31

## Data Availability

Data is contained within the article and supplementary file.

## Supplementary Materials

The following are available online; Tables S1–S10: Comparison of experimental and computed NMR data for compound **3**, **5**, **6**, **7**, **9b**–**9h**; Figures S1–S8: 1H NMR (600 MHz, DMSO) of compound **3**, **7**, **9a**,**b**,**d**,**e**,**g**,**h**; Figures S9–S16: UV-Vis spectrum (calculated at TD-DFT/PBE0/def2TZVPDMSO) of compound **9a**–**h**; Figure S17: Representation of frontier orbitals of the molecules.

## Sample Availability

Not available.
