# Supplementary material for: Synthesis of Functionalized Thiophene Based Pyrazole Amides via Various Catalytic Approaches: Structural Features through Computational Applications and Nonlinear Optical Properties
Source: Molecules. 2022 Jan 7;27(2):360. doi: 10.3390/molecules27020360 (PMC8778362; doi:10.3390/molecules27020360)
Supplement: Supplementary file 1 [file molecules-27-00360-s001.zip › molecules-1516288-supplementary.pdf]

Supplementary Data

Synthesis of functionalized thiophene based pyrazole amides *via* various catalytic approaches: Structural features through Computational applications and Nonlinear optical properties

Iram Kanwal[1], Nasir Rasool[1,*], Syeda Huda Mehdi Zaidi[2], Zainul Amiruddin Zakaria[3,4,*], Muhammad Bilal[1], Muhammad Ali Hashmi[2], Adeel Mubarik[1], Gulraiz Ahmad[1], Syed Adnan Ali Shah[5,6]

[1] Department of Chemistry, Government College, University Faisalabad, 38000, Pakistan; iramchemist117@gmail.com (I.R.), nasirrasool@gcuf.edu.pk (N.R.), muhammadbilalgcuf@gmail.com (M.B.), adeelmubarik2@gmail.com (A.M.), gulchemist35@gmail.com (G.A.)

[2] Department of Chemistry, Division of Science & Technology, University of Education, Lahore, 54770, Pakistan; hudazaidi36@gmail.com (S.H.M.Z), muhammad.hashmi@ue.edu.pk (M.A.H.)

[3] Department of Biomedical Sciences, Faculty of Medicine and Health Sciences, Universiti Malaysia Sabah, Jalan UMS, 88400 Kota Kinabalu, Sabah, Malaysia; drzazakaria@gmail.com (Z.A.Z.)

[4] Halal Product Development Unit, Halal Product Research Institute, Universiti Putra Malaysia, 43400 UPM Serdang, Selangor, Malaysia

[5] Faculty of Pharmacy, Universiti Teknologi MARA Cawangan Selangor Kampus Puncak Alam, Bandar Puncak Alam 42300, Selangor D. E., Malaysia; syedadnan@uitm.edu.my (S.A.A.S)

[6] Atta-ur-Rahman Institute for Natural Products Discovery (AuRIns), Universiti Teknologi MARA Cawangan Selangor Kampus Puncak Alam, Bandar Puncak Alam 42300, Selangor D. E., Malaysia

***** Correspondence: nasirrasool@gcuf.edu.pk , (N.R); Tel.: +92-332-7491790 (N.R.); drzazakaria@gmail.com, (Z.A.Z.); Tel.: +6019-2117090 (Z.A.Z.)

Table of Contents

Table S1: Comparison of Experimental and Computed NMR data for compound 9b 3

Table S2: Comparison of Experimental and Computed NMR data for compound 9c 4

Table S3: Comparison of Experimental and Computed NMR data for compound 9d 5

Table S4: Comparison of Experimental and Computed NMR data for compound 9e 6

Table S5: Comparison of Experimental and Computed NMR data for compound 9g 7

Table S6: Comparison of Experimental and Computed NMR data for compound 9h 8

Table S7: Comparison of Experimental and Computed NMR data for compound 3 9

Table S8: Comparison of Experimental and Computed NMR data for compound 5 10

Table S9: Comparison of Experimental and Computed NMR data for compound 6 10

Table S10: Comparison of Experimental and Computed NMR data for compound 7 11

Figure S1: ^{1}H NMR (600 MHz, DMSO) of compound 3...12

Figure S2: ^{1}H NMR (600 MHz, DMSO) of compound 7...12

Figure S3: ^{1}H NMR (600 MHz, DMSO) of compound 9a...13

Figure S4: ^{1}H NMR (600 MHz, DMSO) of compound 9b. 14

Figure S5: ^{1}H NMR (600 MHz, DMSO) of compound 9d. 15

Figure S6: ^{1}H NMR (600 MHz, DMSO) of compound 9e...16

Figure S7: ^{1}H NMR (600 MHz, DMSO) of compound 9g...17

Figure S8: ^{1}H NMR (600 MHz, DMSO) of compound 9h 17

Figure S9: UV-Vis spectrum (Calculated at TD-DFT/PBE0/def2TZVPDMSO) of compound 9a 18

Figure S10: UV-Vis spectrum (Calculated at TD-DFT/PBE0/def2TZVPDMSO) of compound 9b 18

Figure S11: UV-Vis spectrum (Calculated at TD-DFT/PBE0/def2TZVPDMSO) of compound 9c 19

Figure S12: UV-Vis spectrum (Calculated at TD-DFT/PBE0/def2TZVPDMSO) of compound 9d 19

Figure S13: UV-Vis spectrum (Calculated at TD-DFT/PBE0/def2TZVPDMSO) of compound 9e 20

Figure S14: UV-Vis spectrum (Calculated at TD-DFT/PBE0/def2TZVPDMSO) of compound 9f.................20

Figure S15: UV-Vis spectrum (Calculated at TD-DFT/PBE0/def2TZVPDMSO) of compound 9g 21

Figure S16: UV-Vis spectrum (Calculated at TD-DFT/PBE0/def2TZVPDMSO) of compound 9h 21

Figure S17: Representation of Frontier Orbitals of the Molecules 22

Table S1: Comparison of Experimental and Computed NMR data for compound 9b

Compound **9b**				
Carbon No.	Carbon Type	^{1}H-NMR (δ, ppm) Experimental	^{1}H-NMR (δ, ppm) Computed	Δδ, ppm
2	C	-	-	-
3	CH	8.06	6.56	1.50
4	CH	7.50	7.61	-0.11
5	C	-	-	-
1'N	NH	12.15	9.65	2.50
3'	C	-	-	-
4'	CH	6.37	5.97	0.40
5'	C	-	-	-
5'-Me	CH_3	2.23	2.19	0.04
1''	C	-	-	-
2''	CH	7.72	8.21	-0.49
3''	CH	7.89	8.33	-0.44
4''	C	-	-	-
4''-COMe	CH_3	3.88	3.72	0.16
5''	CH	7.89	8.47	-0.58
6''	CH	7.72	8.10	-0.38
Mean Absolute Error (MAE) = 0.28				
Root Mean Square Error (RMSE) = 0.63				

Table S2: Comparison of Experimental and Computed NMR data for compound 9c

Compound **9c**				
Carbon No.	Carbon Type	^{1}H-NMR (δ, ppm) Experimental	^{1}H-NMR (δ, ppm) Computed	Δδ, ppm
2	C	-	-	-
3	CH	8.06	6.45	1.61
4	CH	7.50	7.31	0.19
5	C	-	-	-
1'N	NH	12.12	9.66	2.46
3'	C	-	-	-
4'	CH	6.36	6.00	0.36
5'	C	-	-	-
5'-Me	CH_3	2.23	2.20	0.03
1''	C	-	-	-
2''	CH	7.26	7.93	-0.67
3''	CH	7.62	7.11	0.51
4''	C	-	-	-
4''-OMe	CH_3	2.37	3.71	-1.34
5''	CH	7.62	7.21	0.41
6''	CH	7.26	8.13	-0.87

Mean Absolute Error (MAE) = 0.35

Root Mean Square Error (RMSE) = 0.71

Table S3: Comparison of Experimental and Computed NMR data for compound 9d

Compound **9d**				
Carbon No.	Carbon Type	^{1}H-NMR (δ, ppm) Experimental	^{1}H-NMR (δ, ppm) Computed	Δδ, ppm
2	C	-	-	-
3	CH	8.40	6.52	1.88
4	CH	7.70	7.52	0.18
5	C	-	-	-
1'N	NH	12.14	9.66	2.48
3'	C	-	-	-
4'	CH	6.37	5.99	0.38
5'	C	-	-	-
5'-Me	CH_3	2.23	2.20	0.03
1"	C	-	-	-
2"	CH	7.98	8.04	-0.06
3"	CH	7.98	7.54	0.44
4"	C	-	-	-
4"-COMe	CH_3	2.66	2.35	0.31
5"	CH	7.98	7.71	0.27
6"	CH	7.98	7.96	0.02
Mean Absolute Error (MAE) = 0.25				
Root Mean Square Error (RMSE) = 0.65				

Table S4: Comparison of Experimental and Computed NMR data for compound 9e

Compound **9e**				
Carbon No.	Carbon Type	^{1}H-NMR (δ, ppm) Experimental	^{1}H-NMR (δ, ppm) Computed	Δδ, ppm
2	C	-	-	-
3	CH	8.04	7.34	0.70
4	CH	7.89	7.45	0.44
5	C	-	-	-
1'N	NH	12.14	9.18	2.96
3'	C	-	-	-
4'	CH	6.35	5.71	0.64
5'	C	-	-	-
5'-Me	CH_3	2.22	2.11	0.11
2''	CH	7.67	7.96	-0.29
3''	C	-	-	-
4''	CH	7.43	7.89	-0.46

Mean Absolute Error (MAE) = 0.24

Root Mean Square Error (RMSE) = 0.65

Table S5: Comparison of Experimental and Computed NMR data for compound 9g

Compound **9g**				
Carbon No.	Carbon Type	^{1}H-NMR (δ, ppm) Experimental	^{1}H-NMR (δ, ppm) Computed	Δδ, ppm
2	C	-	-	-
3	CH	8.05	6.69	1.36
4	CH	7.50	7.43	0.07
5	C	-	-	-
1'N	NH	12.12	9.51	2.61
3'	C	-	-	-
4'	CH	6.36	5.87	0.49
5'	C	-	-	-
5'-Me	CH_3	2.23	2.15	0.08
1''	C	-	-	-
2''	CH	7.34	7.78	-0.44
3''	C	-	-	-
3''-Me	CH_3	2.32	2.32	0.00
4''	CH	7.02	7.29	-0.27
5''	C	-	-	-
5''-Me	CH_3	2.32	2.33	-0.01
6''	CH	7.34	7.68	-0.34
Mean Absolute Error (MAE) = 0.24				
Root Mean Square Error (RMSE) = 0.62				

Table S6: Comparison of Experimental and Computed NMR data for compound 9h

Compound **9h**				
Carbon No.	Carbon Type	^{1}H-NMR (δ, ppm) Experimental	^{1}H-NMR (δ, ppm) Computed	Δδ, ppm
2	C	-	-	-
3	CH	8.09	6.67	1.42
4	CH	7.40	7.57	-0.17
5	C	-	-	-
1'N	NH	12.15	9.54	2.61
3'	C	-	-	-
4'	CH	6.36	5.88	0.48
5'	C	-	-	-
5'-Me	CH_3	2.23	2.16	0.07
1''	C	-	-	-
2''	CH	7.26	7.72	-0.46
3''	C	-	-	-
4''	CH	6.98	6.97	0.01
5''	C	-	-	-
6''	CH	7.11	7.48	-7.48

Mean Absolute Error (MAE) = 0.53

Root Mean Square Error (RMSE) = 2.19

Table S7: Comparison of Experimental and Computed NMR data for compound 3

Compound **3**				
Carbon No.	Carbon Type	^{1}H-NMR (δ, ppm) Experimental	^{1}H-NMR (δ, ppm) Computed	Δδ, ppm
2	C	-	-	-
3	CH	7.32	6.53	0.79
4	CH	7.67	7.02	0.65
5	C	-	-	-
3'	C	-	-	-
3'-Me	CH_3	2.25	2.24	4.03
4'	CH	6.27	2.24	-2.24
5'	C	-	-	-
1''	C	-	-	-
2''	CH	7.50	7.95	-0.45
3''	CH	7.44	7.72	-0.28
4''	CH	7.34	7.70	-0.36
5''	CH	7.44	7.89	-0.45
6''	CH	7.50	7.97	-0.53

Mean Absolute Error (MAE) = 0.42

Root Mean Square Error (RMSE) = 0.98

Table S8: Comparison of Experimental and Computed NMR data for compound 5

Compound **5**				
Carbon No.	Carbon Type	^{1}H-NMR (δ, ppm) Experimental	^{1}H-NMR (δ, ppm) Computed	Δδ, ppm
1 N-COOC(Me)$_3$	$(CH_3)_3$	1.37	1.40	-0.03
3	C	-	-	0.00
3-NH_2	NH_2	6.01	3.94	2.07
4	CH	6.52	5.70	0.82
5	C	-	-	0.00
5-Me	CH_3	2.46	2.26	0.20
Mean Absolute Error (MAE) = 0.13				
Root Mean Square Error (RMSE) = 0.45				

Table S9: Comparison of Experimental and Computed NMR data for compound 6

Compound **6**				
Carbon No.	Carbon Type	^{1}H-NMR (δ, ppm) Experimental	^{1}H-NMR (δ, ppm) Computed	Δδ, ppm
2	C	-	-	-
3	CH	7.91	7.42	0.49
4	CH	7.31	7.13	0.18
5	C	-	-	-
1 N'-COOCMe$_3$	$(CH_3)_3$	1.94	1.45	0.49
3'	C	-	-	-
4'	CH	6.32	5.73	0.59
5'	C	-	-	-
5'-Me	CH_3	2.22	2.25	-0.03
Mean Absolute Error (MAE) = 0.17				
Root Mean Square Error (RMSE) = 0.49				

Table S10: Comparison of Experimental and Computed NMR data for compound 7

Compound **7**				
Carbon No.	Carbon Type	^{1}H-NMR (δ, ppm) Experimental	^{1}H-NMR (δ, ppm) Computed	Δδ, ppm
2	C	-	-	-
3	CH	7.90	6.45	1.45
4	CH	7.30	6.93	0.37
5	C	-	-	-
1'N	NH	12.15	9.63	2.52
3'	C	-	-	-
4'	CH	6.32	5.94	0.38
5'	C	-	-	-
5'-Me	CH_3	2.21	2.18	0.03
Mean Absolute Error (MAE) = 0.28				
Root Mean Square Error (RMSE) = 0.74				

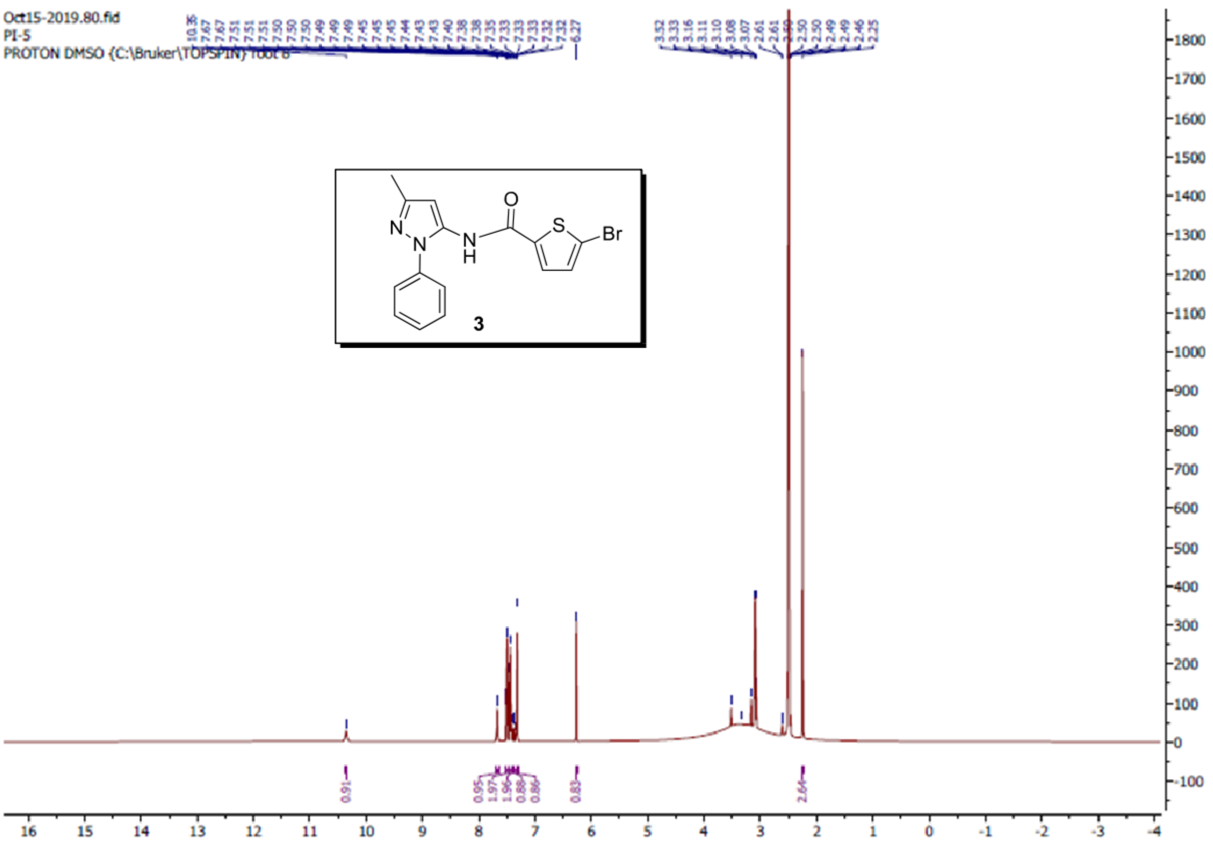

Figure S1: ^{1}H NMR (600 MHz, DMSO) of compound 3.

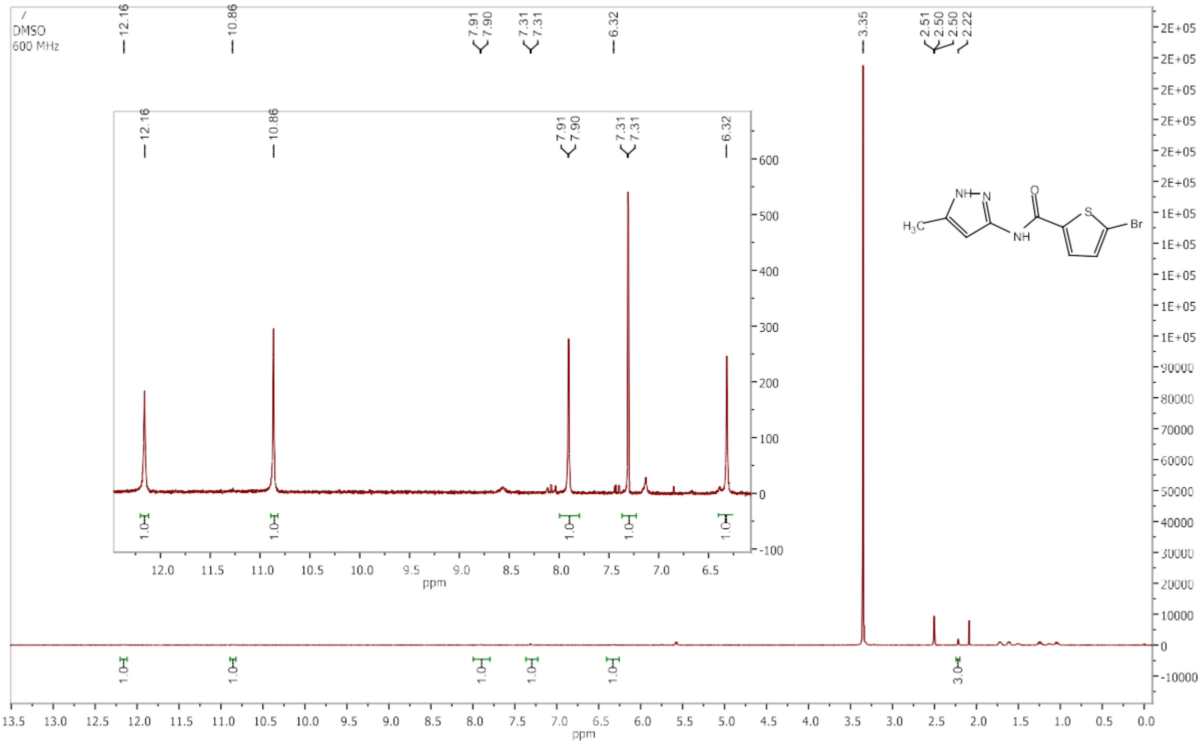

Figure S2: ^{1}H NMR (600 MHz, DMSO) of compound 7

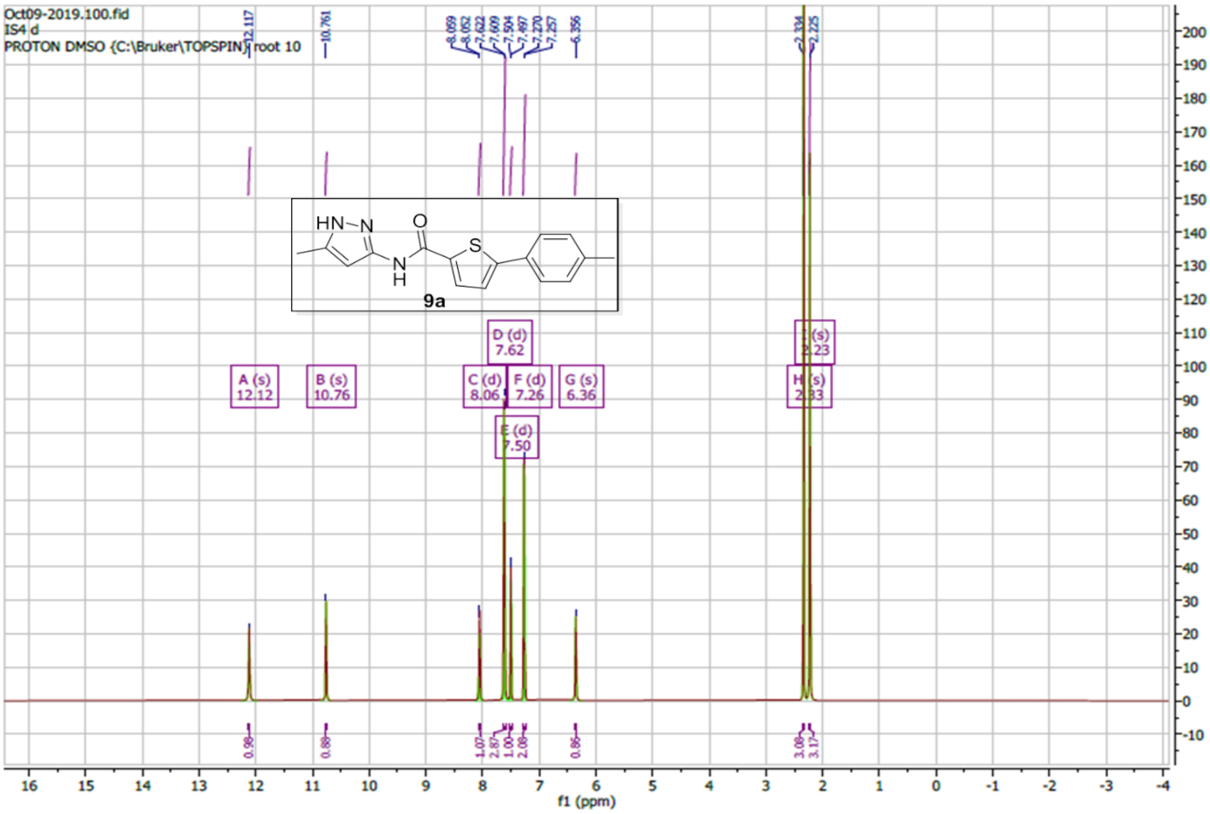

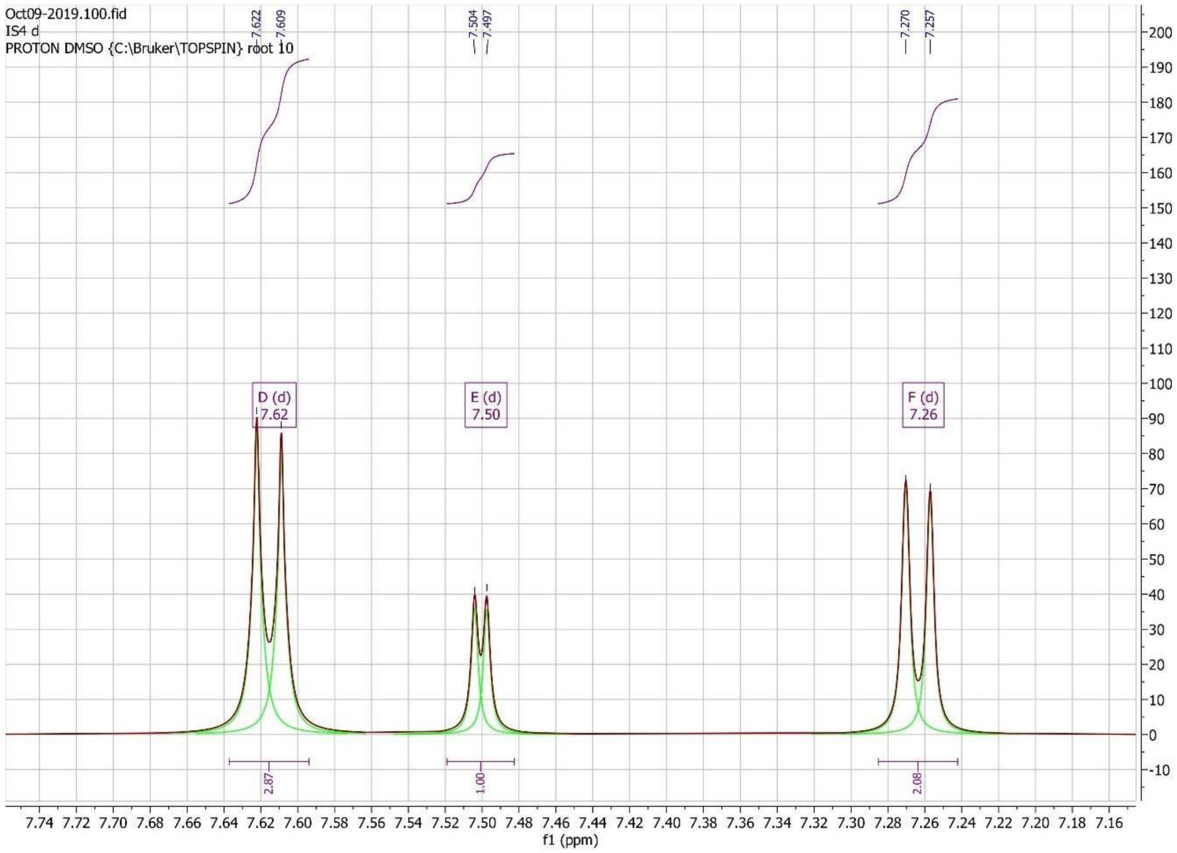

Figure S3: ^{1}H NMR (600 MHz, DMSO) of compound 9a.

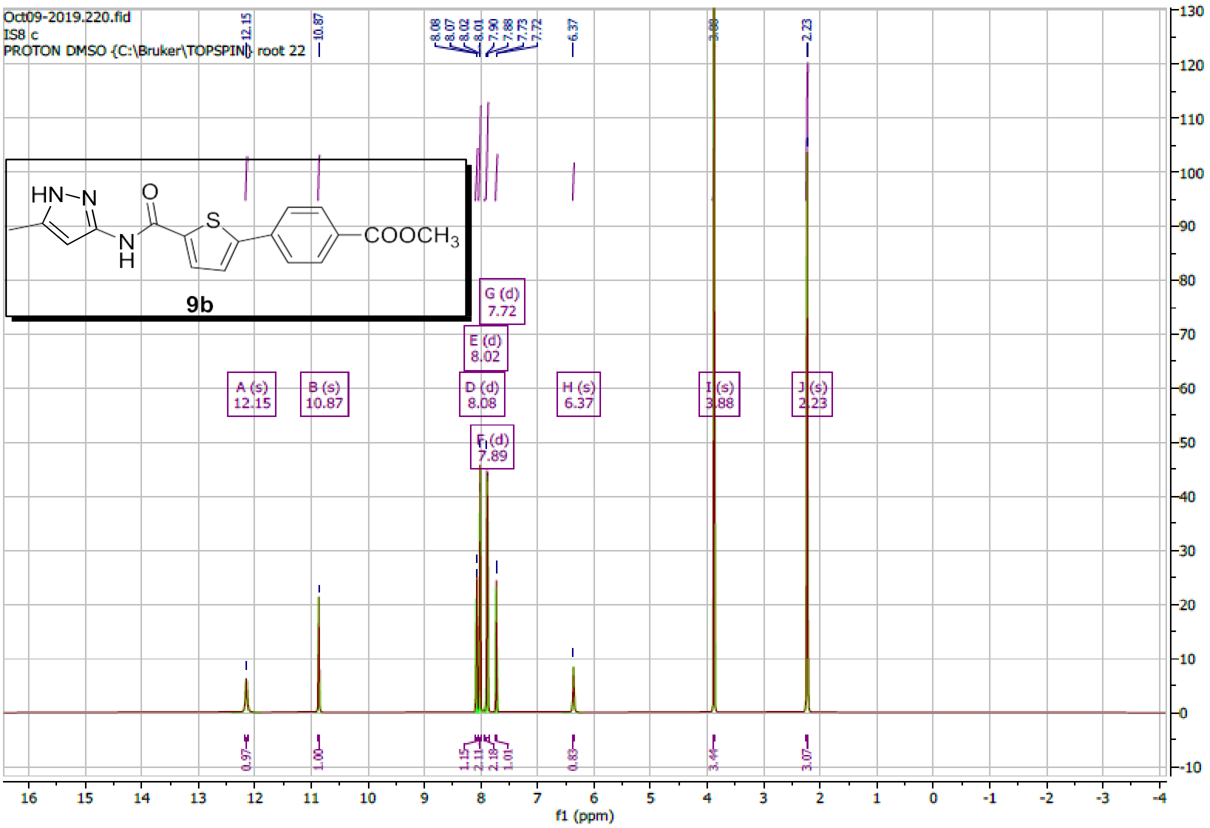

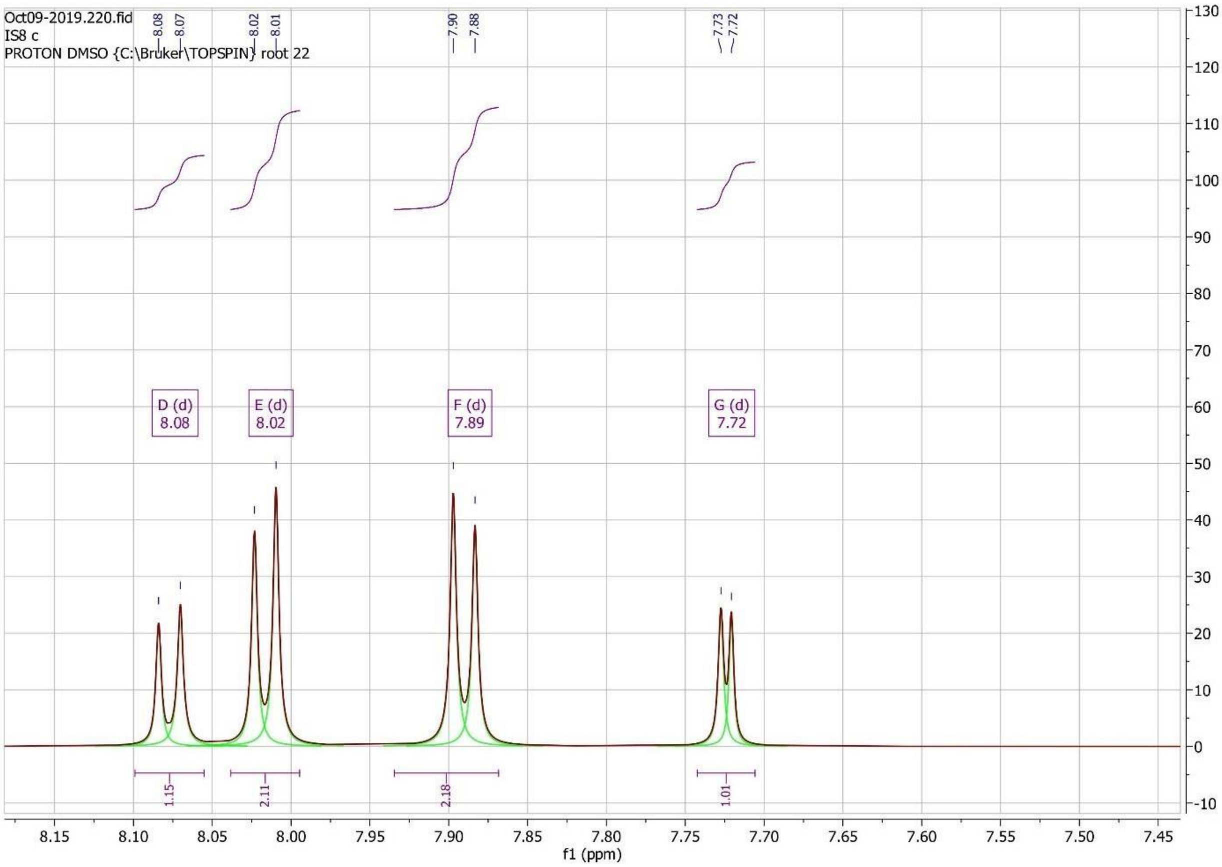

Figure S4: ^{1}H NMR (600 MHz, DMSO) of compound 9b.

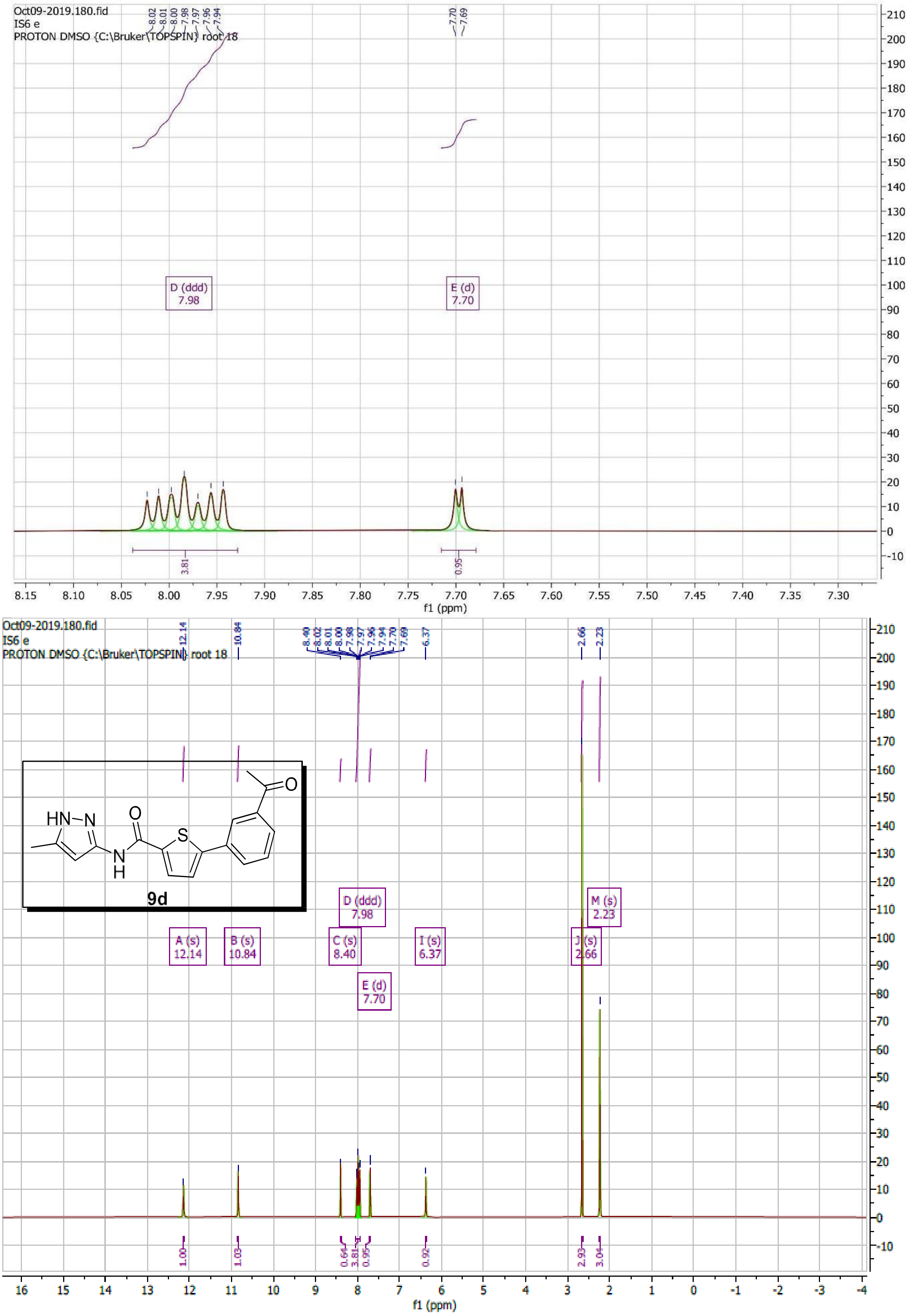

Figure S5: ^{1}H NMR (600 MHz, DMSO) of compound 9d.

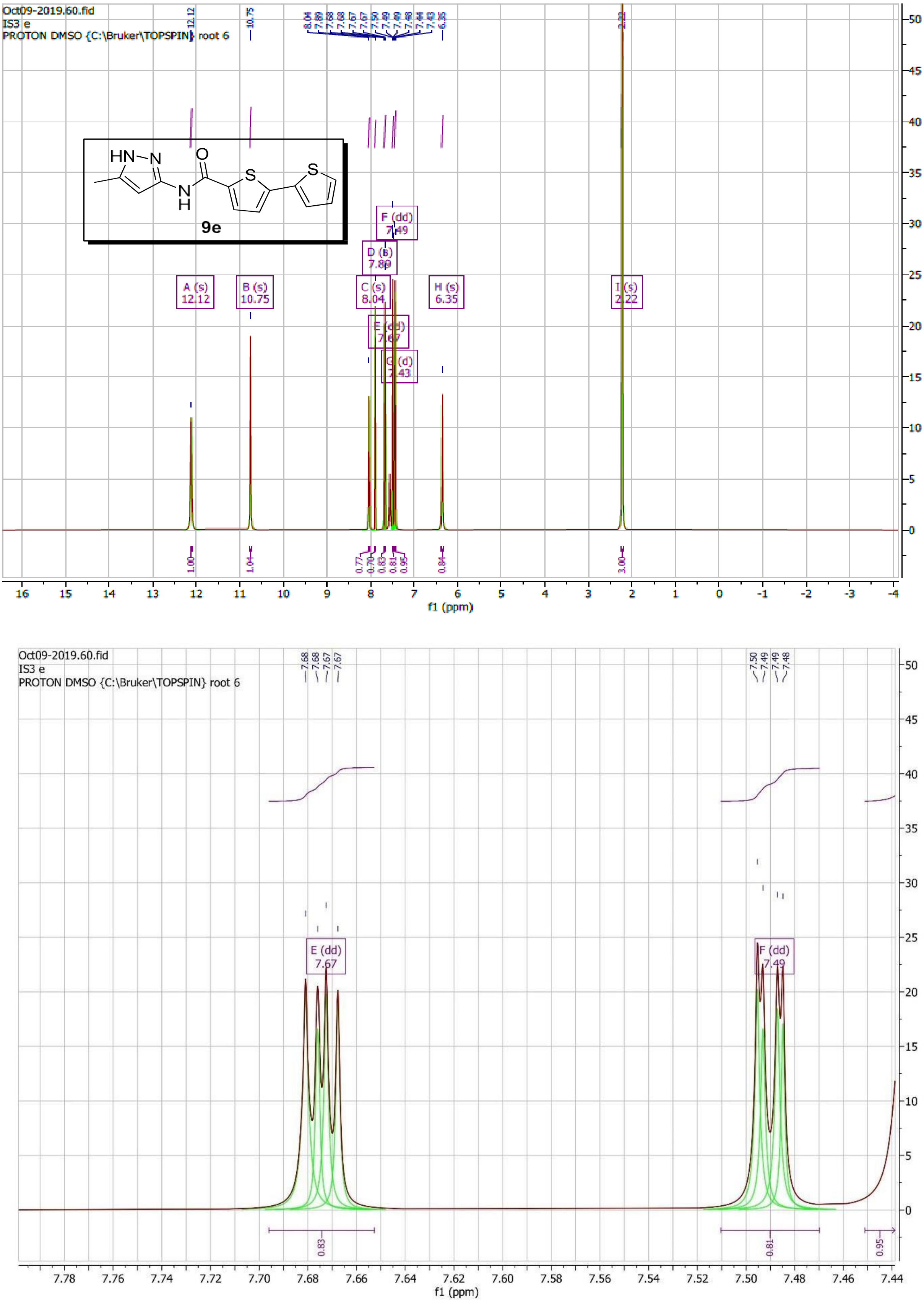

Figure S6: ^{1}H NMR (600 MHz, DMSO) of compound 9e

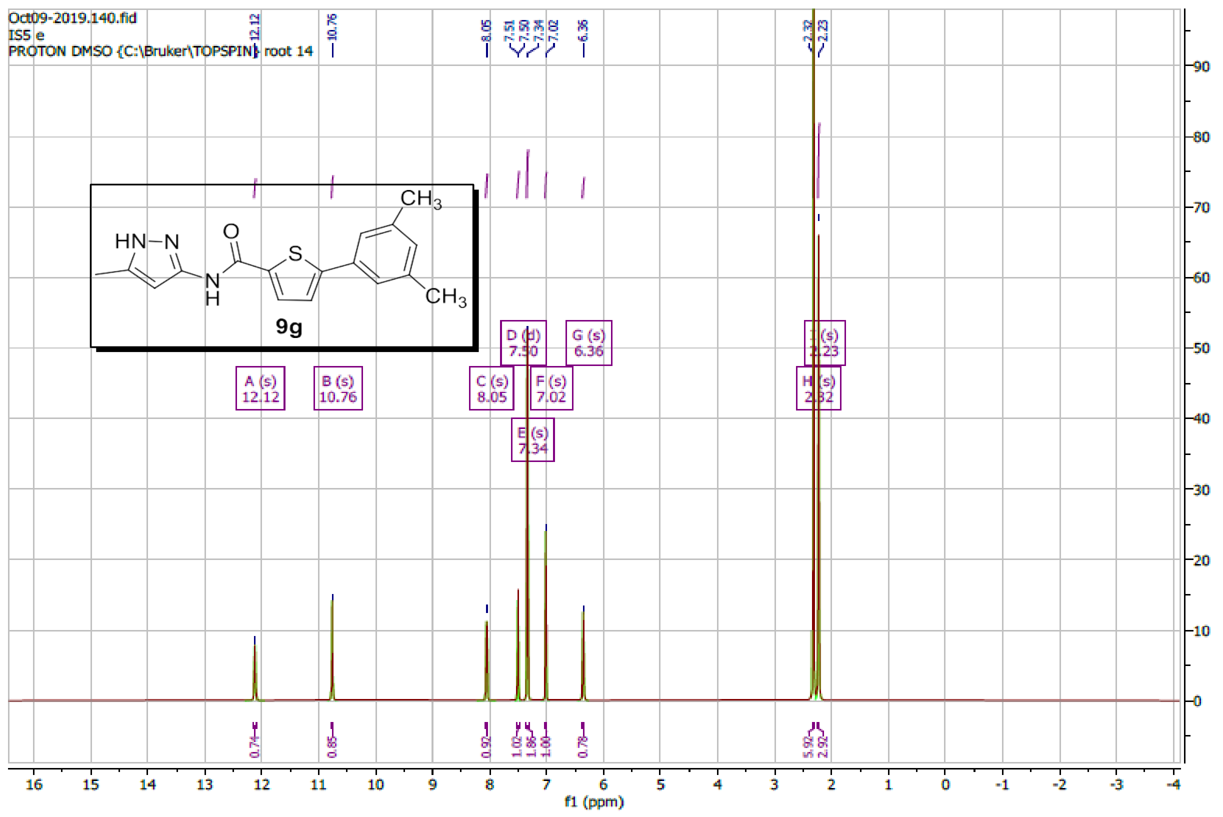

Figure S7: ^{1}H NMR (600 MHz, DMSO) of compound 9g.

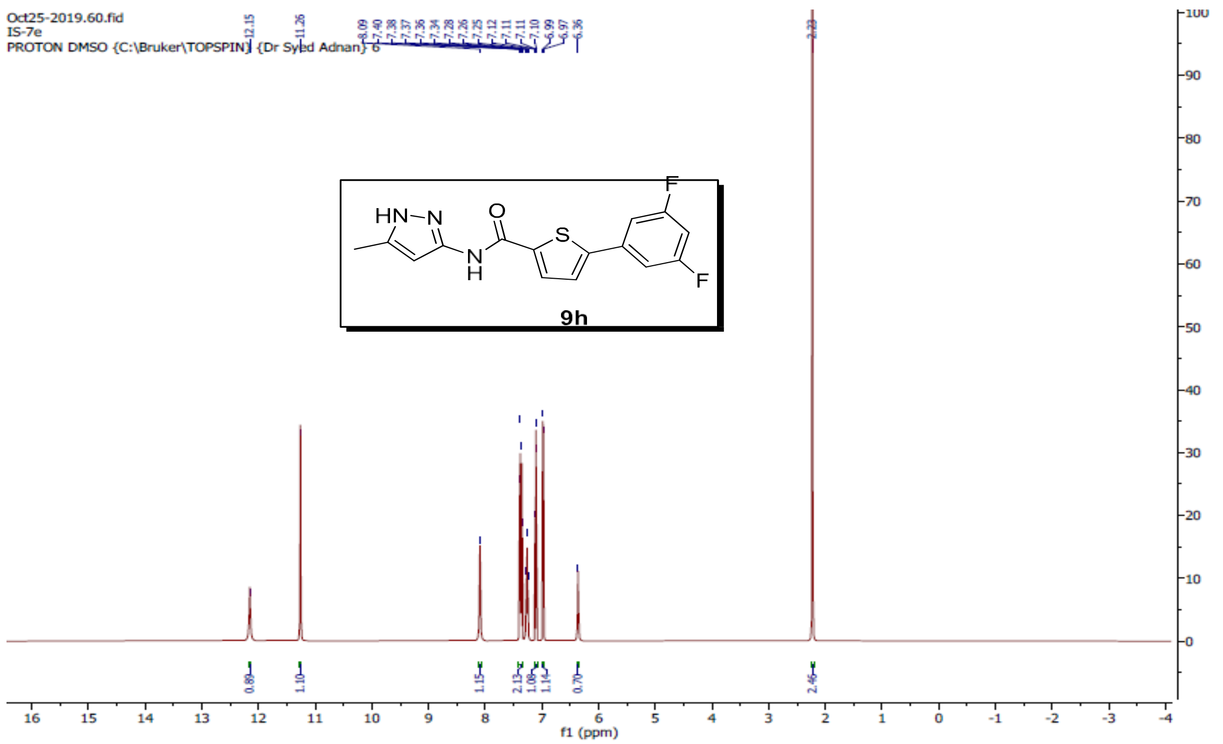

Figure S8: ^{1}H NMR (600 MHz, DMSO) of compound 9h

.

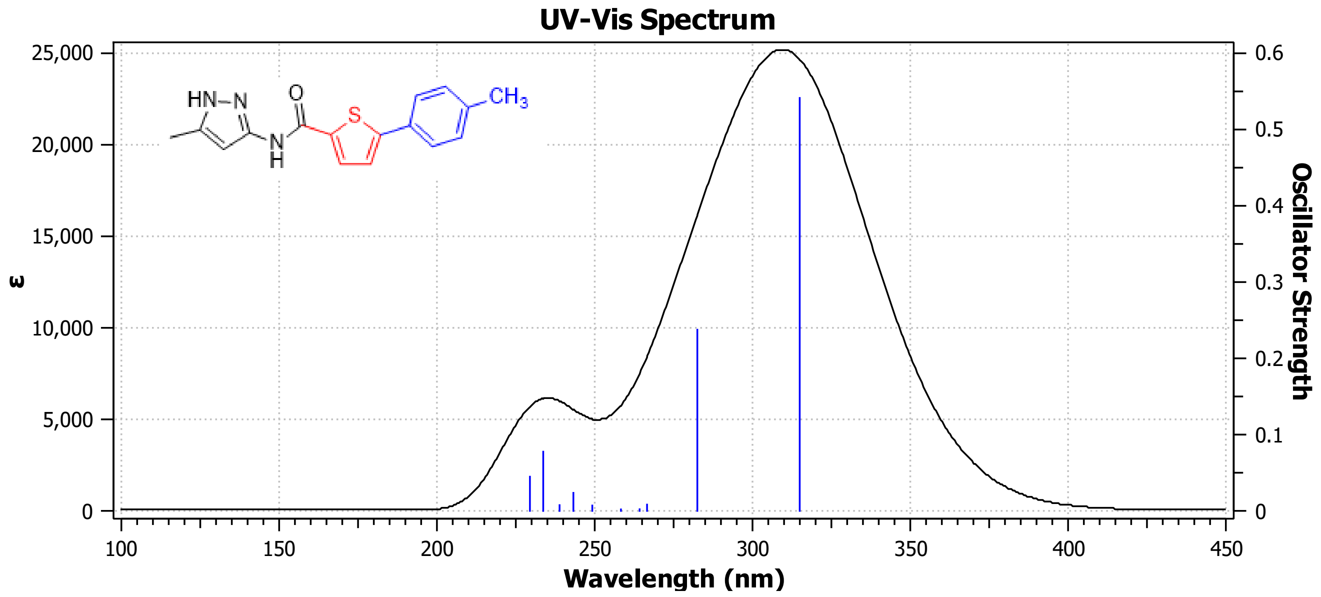

Figure S9: UV-Vis spectrum (Calculated at TD-DFT/PBE0/def2TZVPDMSO) of compound 9a

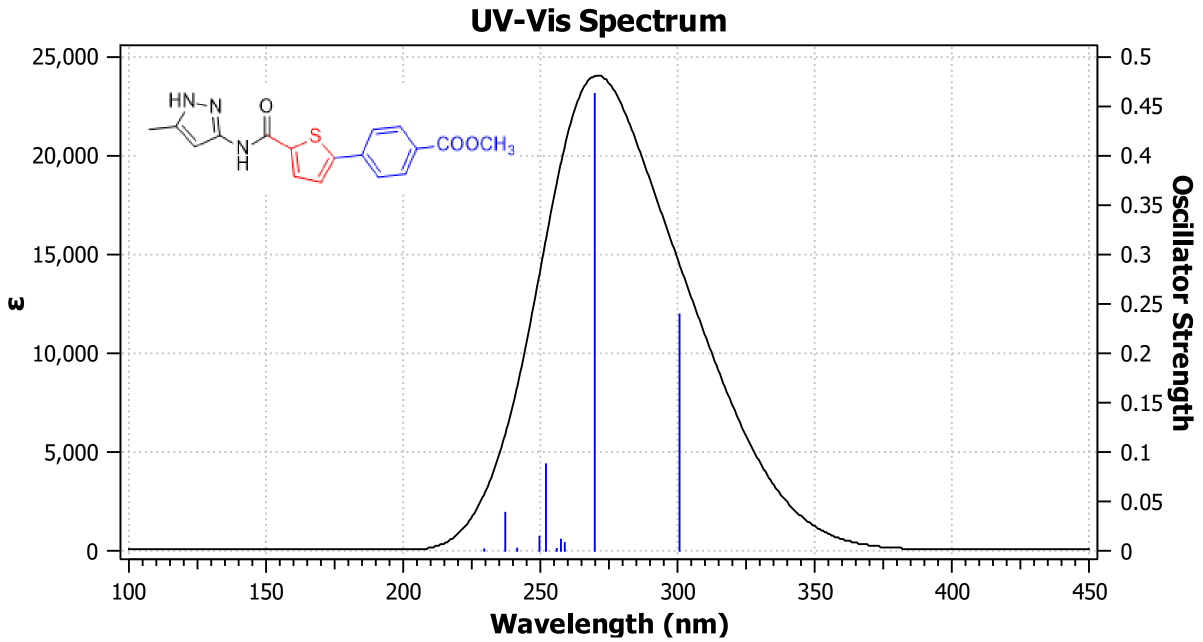

Figure S10: UV-Vis spectrum (Calculated at TD-DFT/PBE0/def2TZVPDMSO) of compound 9b

Compound 9c

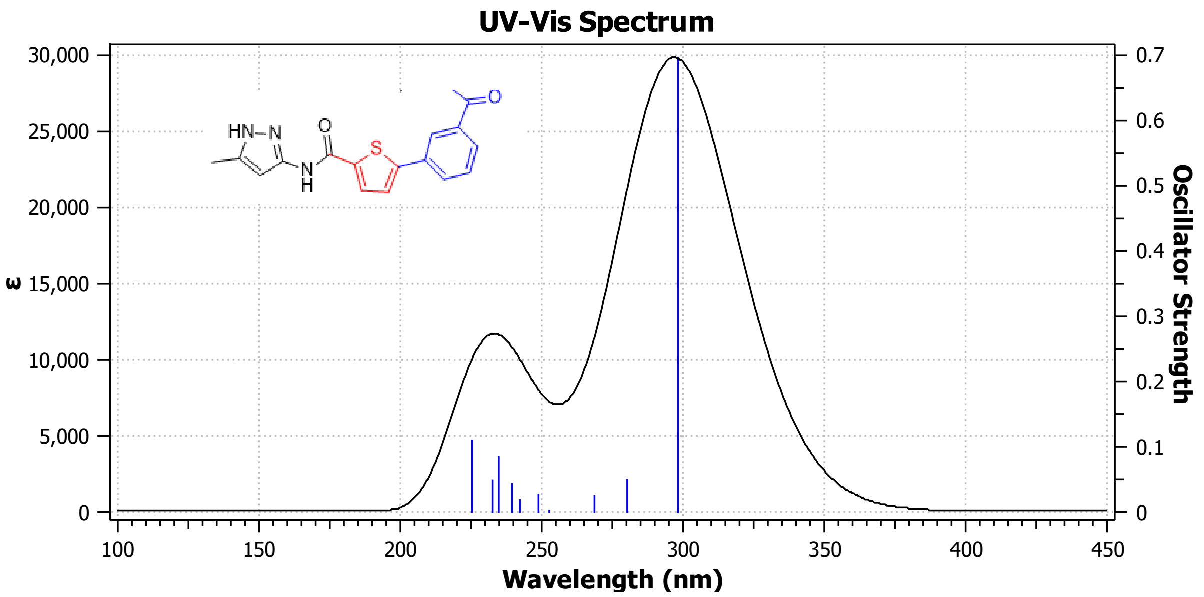

Figure S11: UV-Vis spectrum (Calculated at TD-DFT/PBE0/def2TZVPDMSO) of compound 9c

Compound 9d

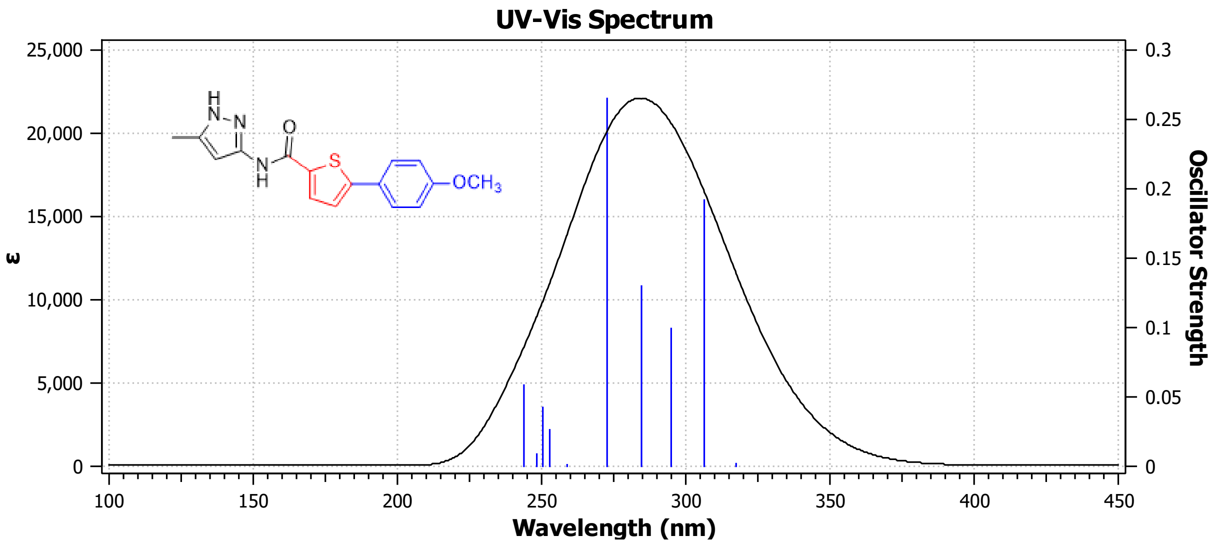

Figure S12: UV-Vis spectrum (Calculated at TD-DFT/PBE0/def2TZVPDMSO) of compound 9d

Compound 9e

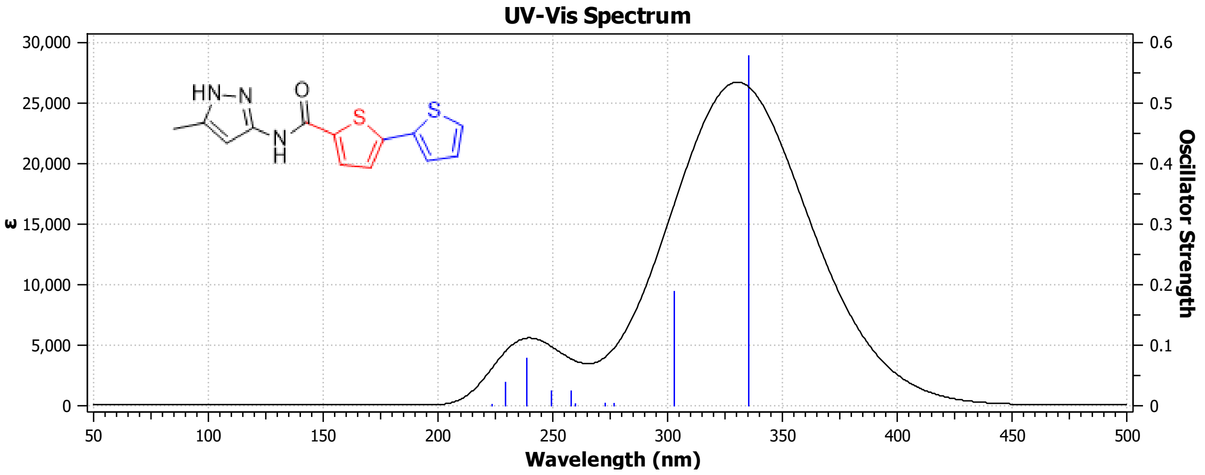

Figure S13: UV-Vis spectrum (Calculated at TD-DFT/PBE0/def2TZVPDMSO) of compound 9e

Compound 9f

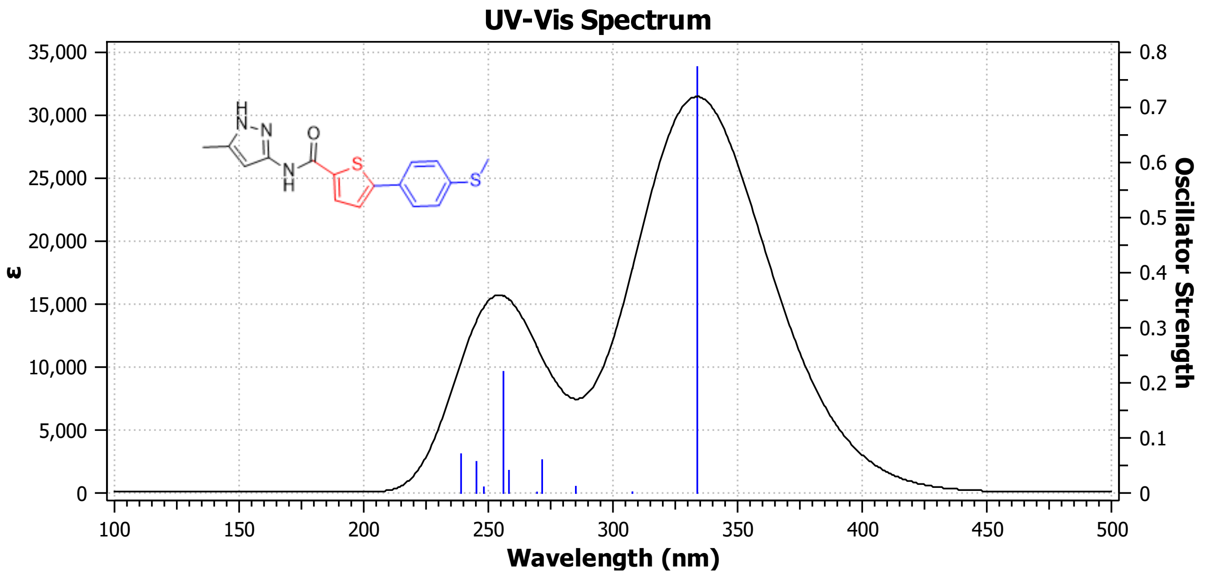

Figure S14: UV-Vis spectrum (Calculated at TD-DFT/PBE0/def2TZVPDMSO) of compound 9f

Compound 9g

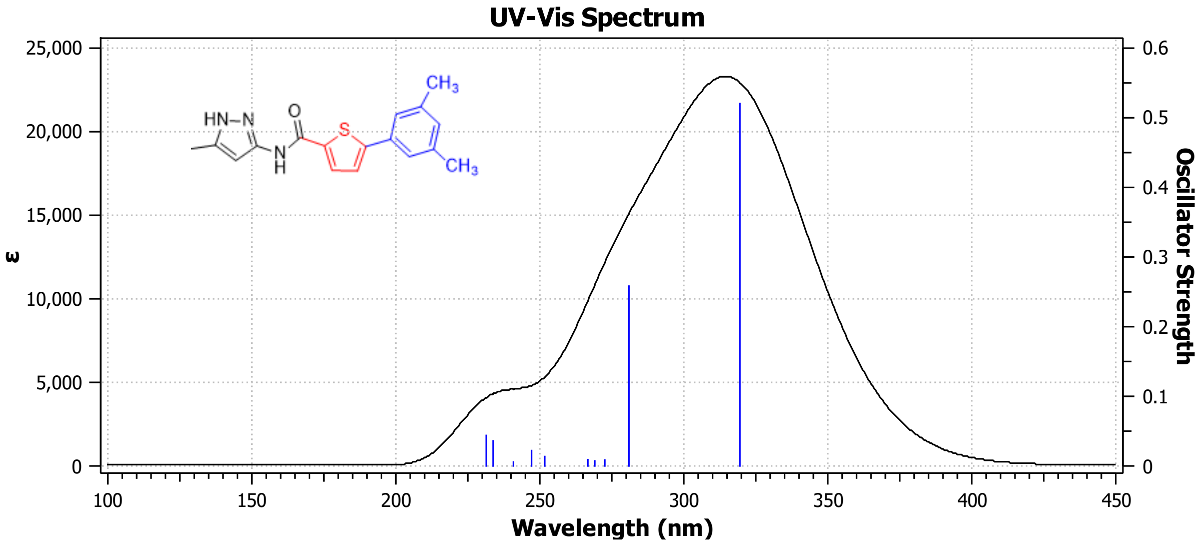

Figure S15: UV-Vis spectrum (Calculated at TD-DFT/PBE0/def2TZVPDMSO) of compound 9g

Compound 9h

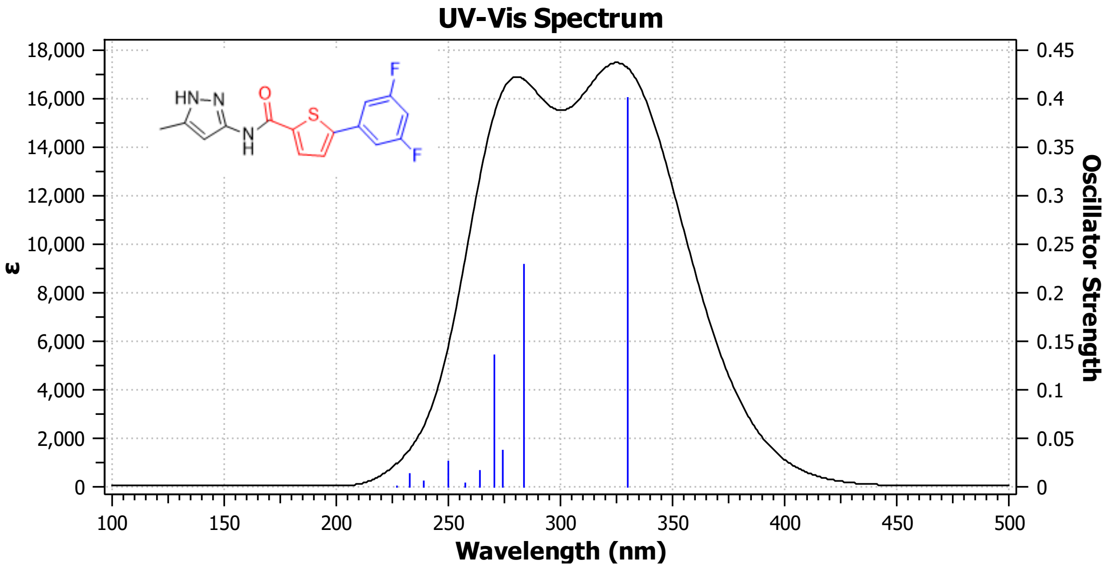

Figure S16: UV-Vis spectrum (Calculated at TD-DFT/PBE0/def2TZVPDMSO) of compound 9h

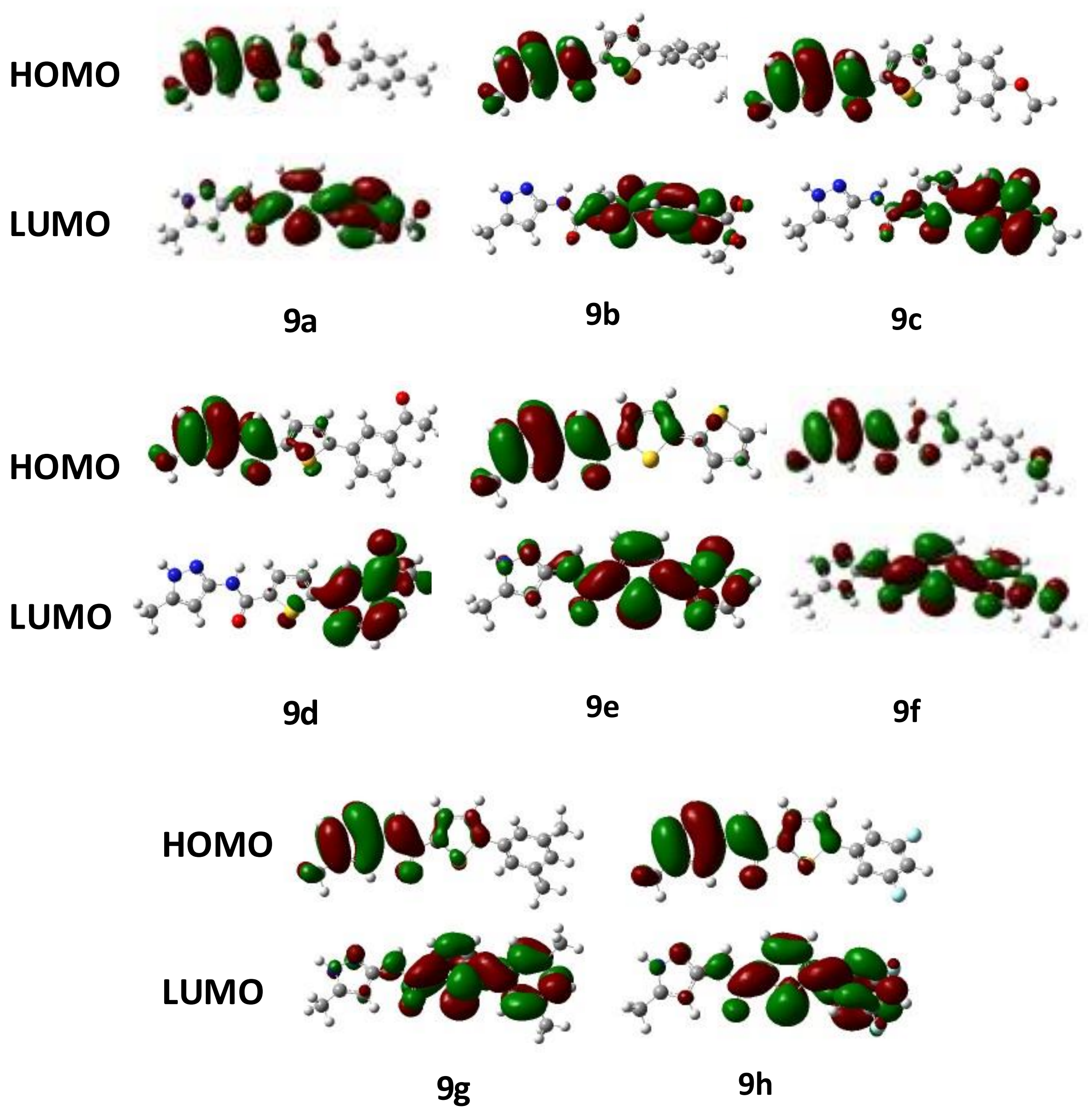

Figure S17: Representation of Frontier Orbitals of the Molecules
